# The Role of Omega-3 Polyunsaturated Fatty Acid Supplementation in Postoperative Recovery of Colorectal Cancer: Systematic Review and Meta-Analysis

**DOI:** 10.3390/nu18010173

**Published:** 2026-01-05

**Authors:** Huzhong Li, Zhenze Xu, Yamin Chen, Jianming Guo, Qihe Wang, Dong Liang, Pengfeng Qu, Taotao Deng, Yuan Yuan, Jiao Xu, Haiqin Fang, Ziyuan Wang

**Affiliations:** 1 National Health Commission Key Laboratory of Food Safety Risk Assessment, China National Center for Food Safety Risk Assessment, Beijing 100022, China; lihuzhong@cfsa.net.cn (H.L.); xuzz_1129@163.com (Z.X.);; 2 Key Laboratory of Geriatric Nutrition and Health, Beijing Technology and Business University, Ministry of Education, Beijing 100048, China; 3 National Center of Technology Innovation for Dairy, Hohhot 010110, China

**Keywords:** Omega-3 polyunsaturated fatty acids, colorectal cancer, meta-analysis, postoperative recovery, clinical nutrition

## Abstract

**Background:** China is currently developing standards for Food for Special Medical Purposes (FSMP) targeting for oncology patients. However, substantial challenges remain in defining optimal fortification levels of omega-3 polyunsaturated fatty acids (ω-3 PUFAs). Accumulating evidence suggests that ω-3 PUFA intake improves postoperative prognosis by modulating oncological parameters in colorectal cancer (CRC) patients. This meta-analysis aimed to evaluate the therapeutic efficacy of ω-3 PUFA supplementation in enhancing postoperative safety and recovery stability following CRC surgery, to address critical gaps in nutritional interventions for optimizing clinical outcomes. These findings are expected to FSMP standard development, clinical nutrition protocols and product innovation. **Methods:** A systematic literature search was conducted, in accordance with PRISMA guidelines, across major databases until June 16, 2025. Data were analyzed using RevMan v5.4 (Cochrane Collaboration). **Results:** Thirty-four randomized controlled trials (RCTs) (*n* = 2889) were included. Compared to controls, the ω-3 PUFAs group showed significantly increased levels of nutritional markers: total protein (*p* < 0.00001), albumin (*p* = 0.001); immunological parameters: CD3^+^/CD4^+^/CD8^+^ T-cells, CD4^+^/CD8^+^ ratio (all *p* < 0.0001); Karnofsky Performance Status (KPS) scores (*p* = 0.04); and serum ω-3 PUFA concentrations (*p* = 0.0004). Significant reductions were observed in inflammatory markers, such as procalcitonin, C-reactive protein (CRP), interleukin-6 (IL-6), tumor necrosis factor-α (TNF-α) (*p* = 0.004 to < 0.00001); and clinical outcomes, such as hospitalization duration (*p* < 0.00001), infectious complications (*p* < 0.00001), anastomotic leakage (*p* = 0.0005), surgical site infections (*p* = 0.03). No significant intergroup differences were detected for white blood cells, transcription factor activity, mortality, or crypt cell proliferation indices (*p* = 0.06–0.55). **Conclusions:** Overall, ω-3 PUFA supplementation significantly attenuates postoperative inflammation, enhances immune function, shortens hospitalization, and improves the quality of life in CRC patients, though without mortality benefit. Notably, post hoc dose–response analysis identified a supplementation range of 0.16–0.30 g/kg/day as a potentially optimal supplementation range for Chinese CRC populations, providing foundational evidence for clinical practice and FSMP standardization.

## 1. Introduction

Cancer remains a major threat to global health. Projections indicate that the worldwide cancer incidence (excluding basal cell carcinoma) will reach 28.4 million new cases by 2040, representing a 47% compared with 2020 estimates [1]. Colorectal cancer (CRC) ranks as the third most frequently diagnosed malignancy in men and the second most frequently diagnosed in women worldwide [2]. In 2020, CRC accounted for 1.88 million new diagnoses (1.15 million colon; 0.73 million rectal cancers) and caused approximately 915,800 deaths, making it the second leading cause of cancer-related mortality after lung cancer [1]. The rising global incidence of CRC, particularly among younger individuals, poses substantial challenges for prevention and treatment. Although multimodal therapies combining surgery and chemotherapy have improved survival, prognosis remains heterogeneous due to tumor biology, genetic alterations (KRAS, NRAS and BRAF), and microsatellite instability (MSI) status [3].

ω-3 polyunsaturated fatty acids (ω-3 PUFAs) are essential fatty acids critical for human health [4]. The primary members of this family include eicosapentaenoic acid (EPA), docosahexaenoic acid (DHA), and α-linolenic acid (ALA). ALA, which is derived from plant sources such as flaxseeds and walnuts, differs from the marine-derived EPA and DHA, which are predominantly found in fatty fish, including salmon, mackerel, and sardines [5]. ω-3 PUFAs are sequentially metabolized in the human body through the coordinated actions of desaturases and elongases [6]. In the liver, ALA is first converted by Δ6-desaturase into stearidonic acid (18:4 *n*-3), which is subsequently elongated to eicosatetraenoic acid (20:4 *n*-3) and ultimately converted into EPA. EPA can be further elongated to DPA and then converted to DHA via Δ4-desaturase [7,8]. Notably, in humans, the conversion efficiency of ALA to EPA and DHA is relatively low [8].

Accumulating evidence indicates that ω-3 PUFAs can inhibit the PI3K/AKT signaling pathway, which is a key regulator of cellular proliferation and survival [9]. This mechanism suggests that ω-3 PUFAs may promote apoptotic processes in colorectal carcinoma cells, thereby suppressing tumor progression and metastasis. Furthermore, ω-3 PUFAs have been shown to modulate inflammatory cytokines, notably interleukin-6 (IL-6) and tumor necrosis factor-α (TNF-α), both of which play pivotal roles in carcinogenesis and disease progression, supporting their potential therapeutic application in cancer management [10]. Both clinical and preclinical studies have reported inverse associations between ω-3 PUFA levels and these pro-inflammatory biomarkers [9]. Beyond cancer-related outcomes, ω-3 PUFAs also confer a broad range of additional health benefits. One study demonstrated that ω-3 PUFAs were associated with a reduced risk of gout and rheumatoid arthritis and elucidated the underlying mechanisms, thereby providing a theoretical basis for the dietary prevention of gout and rheumatoid arthritis [11]. Another study reported a significant association between dietary ω-3 PUFAs intake and a slower rate of phenotypic aging, suggesting that a daily intake of approximately 1.1 g may contribute to delayed aging and extend lifespan [12].

Multiple molecular mechanisms have been implicated in the pro-apoptotic effects of ω-3 PUFAs in CRC cells. ω-3 PUFAs regulate cellular redox homeostasis through interconnected pathways, with their antitumor efficacy being closely associated with oxidative stress regulation [13]. These fatty acids induce apoptosis via a series of coordinated mechanisms, including: depolarization of the mitochondrial membrane potential; reactive oxygen species (ROS) generation; activation of caspase-3 and caspase-9; and upregulation of the pro-apoptotic Bax/Bcl-2 ratio. This synergistic cascade elevates intracellular ROS concentrations, enhancing the apoptotic susceptibility of colorectal carcinoma cells [14]. Despite substantial therapeutic advancements in CRC management, dietary interventions involving ω-3 PUFA supplementation represent a promising adjunctive nutritional strategy in clinical oncology [15].

With respect to dietary supplementation of ω-3 PUFAs, multiple countries and international organizations have issued intake recommendations. The World Health Organization (WHO), in its Healthy Diet guidelines, classifies ω-3 PUFAs as an “adequacy component” indicating that sufficient intake is beneficial for health, and recommends that 1–2% of total daily energy intake be consumed as a general target for the prevention of chronic diseases [16]. The UK Department of Health & Social Care recommends an intake of 200 mg/day of combined EPA and DHA for children aged 5 years and older. Nordic countries advise that total ω-3 PUFAs intake should account for at least 1% of total energy intake. In contrast, the U.S. Institute of Medicine (IOM) provides Adequate Intake (AI) values exclusively for ALA, ranging from 0.5 g/day for infants aged 0–6 months to 1.6 g/day for boys aged 14–18 years, without establishing specific intake recommendations for EPA or DHA [17].

The implementation of multidisciplinary tumor therapies has prompted a growing number of randomized controlled trials (RCTs) evaluating ω-3 PUFA interventions for CRC patients. Nevertheless, existing RCTs demonstrate substantial methodological limitations, including small sample sizes and conflicting findings, and few comprehensive meta-analyses have systematically synthesized this evidence. Accordingly, this study employs meta-analytic methods to systematically evaluate the efficacy and safety of ω-3 PUFA supplementation for postoperative recovery among CRC patients.

## 2. Materials and Methods

The study was conducted according to the Preferred Reporting Items for Systematic reviews and Meta-Analyses (PRISMA) 2020 guidelines. To ensure comprehensive and transparent reporting, the PRISMA 2020 checklists for the full text and abstract are provided in the Supplementary Date S1. The protocol for this systematic review and meta-analysis was registered in the International Prospective Register of Systematic Reviews (PROSPERO) under registration number CRD420251246188.

### 2.1. Study Population and Inclusion Criteria

This study enrolled patients with pathologically confirmed CRC across all disease stages. Data from comparative randomized controlled trials (RCTs) were systematically collected. Participants were categorized into two groups according to intervention type:

1. Experimental group: Received ω-3 PUFA supplementation in combination with an isocaloric diet identical to that of the control group;
2. Control group: Received standard dietary intake without any additional nutritional supplementation.

### 2.2. Exclusion Criteria

The following were excluded:

1. Non-English or non-Chinese publications;
2. Non-original research, including reviews, conference abstracts, expert consensus statements, and animal studies;
3. Duplicate publications or non-randomized studies;
4. Studies with unavailable or unreliable data.

### 2.3. Outcome Measures

Primary outcomes included postoperative quality of life (QoL) improvement rate and changes in serological tumor biomarkers in CRC patients (Table 1). Secondary outcomes encompassed other efficacy endpoints, such as clinical response rate, with a comprehensive assessment of all measurable indices beyond the predefined primary outcomes.

### 2.4. Literature Search Strategy

Two independent investigators performed study selection, data extraction, and quality assessment. Any discrepancies were resolved through consultation with a third senior researcher.

A comprehensive search was conducted in the following electronic databases from inception to 16 June 2025: PubMed, Embase, Web of Science, the Cochrane Library, CNKI, WanFang Data, VIP, and CBM. The search strategy utilized a combination of Medical Subject Headings (MeSH) and free-text keywords. The specific search strategy can be seen in the Supplementary Date S2.

The Chinese search terms were translated using a standardized back-translation procedure to ensure conceptual accuracy and were manually reviewed.

Chinese terms: Colon cancer, colon cancer, colon cancer, colorectal cancer, colorectal cancer, *n*-3 fatty acids, *n*-3 unsaturated fatty acids, ω-3 fatty acids, ω-3 unsaturated fatty acids, Omega-3 fatty acids, Omega-3 unsaturated fatty acids, ω-3 fish oil, eicosapentaenoic acid, docosahexaenoic acid, randomized, randomized, RCT in Chinese.

English terms: “Colonic Neoplasms” [MeSH], “Colorectal Neoplasms” [MeSH], “Fatty Acids, Omega-3” [MeSH], “Fish Oils” [MeSH], “Docosahexaenoic Acids” [MeSH], “Eicosapentaenoic Acid” [MeSH], “*n*-3 Fatty Acids”, “Omega-3 Fatty Acids”, “randomized controlled trial” [Publication Type].

### 2.5. Quality Assessment of Included Studies

The methodological quality of the included randomized controlled trials (RCTs) was evaluated using the Cochrane Risk of Bias tool, which assesses the following domains: (1) Random sequence generation; (2) Allocation concealment; (3) Blinding of participants and personnel (performance bias); (4) Incomplete outcome data (attrition bias); (5) Selective reporting (reporting bias); (6) Other potential sources of bias.

### 2.6. Statistical Method

All statistical analyses were performed using RevMan software (v5.4, The Cochrane Collaboration). Risk ratios (RR) with 95% confidence intervals (CIs) were calculated for dichotomous outcomes. Heterogeneity was evaluated using the χ^2^ test and quantified with the *I*^2^ statistic, with the following interpretation: low heterogeneity: *I*^2^ ≤ 50% accompanied by χ^2^ test *p*-value > 0.1; Substantial heterogeneity: *I*^2^ > 50% or χ^2^ test *p*-value ≤ 0.1. When substantial heterogeneity was detected, sensitivity analyses were conducted by omitting each study in turn (the leave-one-out method). Continuous outcomes were analyzed by calculating mean differences (MDs) and their 95% confidence intervals (CIs). Statistical significance was set at *p* < 0.05 for all meta-analyses. A random-effects model was employed when *I*^2^ exceeded 50%. Missing data were handled using interpolation.

### 2.7. Ethics Review

All included studies passed the ethical review. As studies involved minimal risk and used pre-existing anonymized data. Data access complied with institutional data protection policies.

### 2.8. Exploration of the Optimal Dose

To determine the optimal daily intake of omega-3 PUFAs for postoperative recovery in CRC patients, a dose-ranging analysis was performed on data extracted from the 21 RCTs conducted in Chinese populations (Table 2). Dosages were standardized to grams per kilogram per day (g/kg/day). For studies that did not report individual body weights, the mean baseline weight of the cohort was applied. To enable comparison across studies with substantial heterogeneity in treatment duration, assessment methodologies, and patient demographics, outcome measures were compared based on their relative change from baseline.

## 3. Results

### 3.1. Literature Screening Process and Results

**Figure 1:**
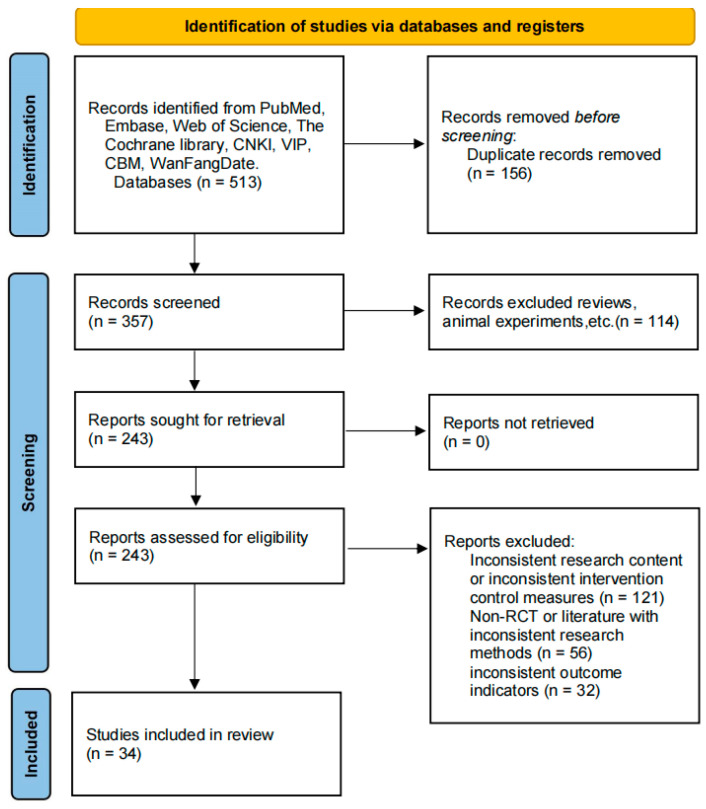
Flow chart of literature screening of the study.

From the initial 513 records retrieved through database searching, 34 randomized controlled trials (RCTs) [16,17,18,19,20,21,22,23,24,25,26,27,28,29,30,31,32,33,34,35,36,37,38,39,40,41,42,43,44,45,46,47,48,49,50,51,52] met the predefined inclusion criteria and were included in the qualitative synthesis. These studies involved a combined cohort of 2889 patients. The complete literature selection process, including reasons for exclusion at each stage, is illustrated in the PRISMA flowchart (Figure 1).

### 3.2. Basic Characteristics of Included Studies

The baseline characteristics of the 34 included studies (21 Chinese and 13 international publications) are summarized in Table 1. The data extraction covered the first author, year of publication, study design, sample size (with mean age and gender distribution), intervention details (duration, dosage, and administration route), and all reported outcome measures.

### 3.3. Results of Bias Risk

**Figure 2:**
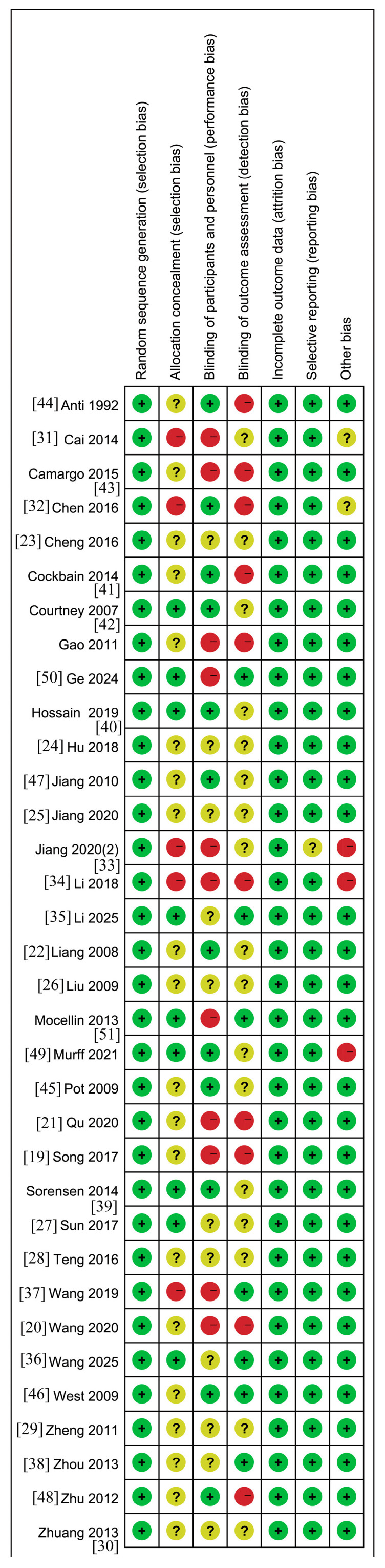
Risks of bias among include studies. Note: Green “+” symbols indicate a low risk of bias, suggesting that the study employed adequate methodology, provided clear reporting, and exhibited a low risk of bias in the corresponding domain. Red “−” symbols denote a high risk of bias, indicating the presence of significant methodological limitations or inappropriate handling in that domain, which may substantially influence the study results. Yellow “?” symbols represent an unclear risk of bias, meaning that the information reported was insufficient to permit a definitive judgment regarding the risk of bias in that domain.

**Figure 3:**
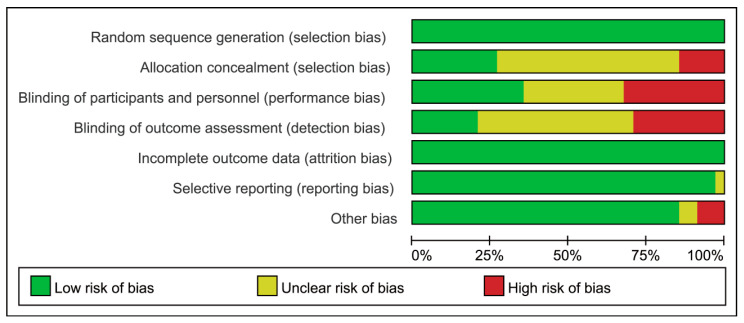
Risk of bias assessment for all included studies. Note: Green (Low risk of bias): Indicates that the study was judged to be at low risk of bias in the corresponding domain according to the Cochrane Risk of Bias tool. Yellow (Unclear risk of bias): Indicates that there was insufficient information to permit a clear judgment of the risk of bias in the corresponding domain. Red (High risk of bias): Indicates that the study was judged to be at high risk of bias in the corresponding domain due to methodological limitations.

The methodological quality of the included studies was evaluated using the Cochrane risk-of-bias tool, with the results summarized in Figure 2 and Figure 3. Among the 34 RCTs, only 9 studies clearly described the method of random sequence generation (e.g., random number table), while the remaining studies did not specify how randomization was achieved. Regarding blinding, 12 studies reported using a double-blind design for both participants and personnel, 11 studies explicitly stated that double-blinding was not implemented, and the blinding procedure was unclear in the remaining 11 studies. One study mentioned a triple-blind design (blinding of participants, personnel, and outcome assessors), while the majority either did not mention or confirmed not using this approach. All included studies were judged to have a low risk of bias concerning incomplete outcome data and selective reporting. Baseline characteristics, including age and gender, were reported and deemed comparable (*p* > 0.05) across all studies. It is noteworthy that one study was assessed as having a high risk of bias in other domains.

### 3.4. Meta-Analysis Results

#### 3.4.1. Blood Indicators

**Figure 4:**
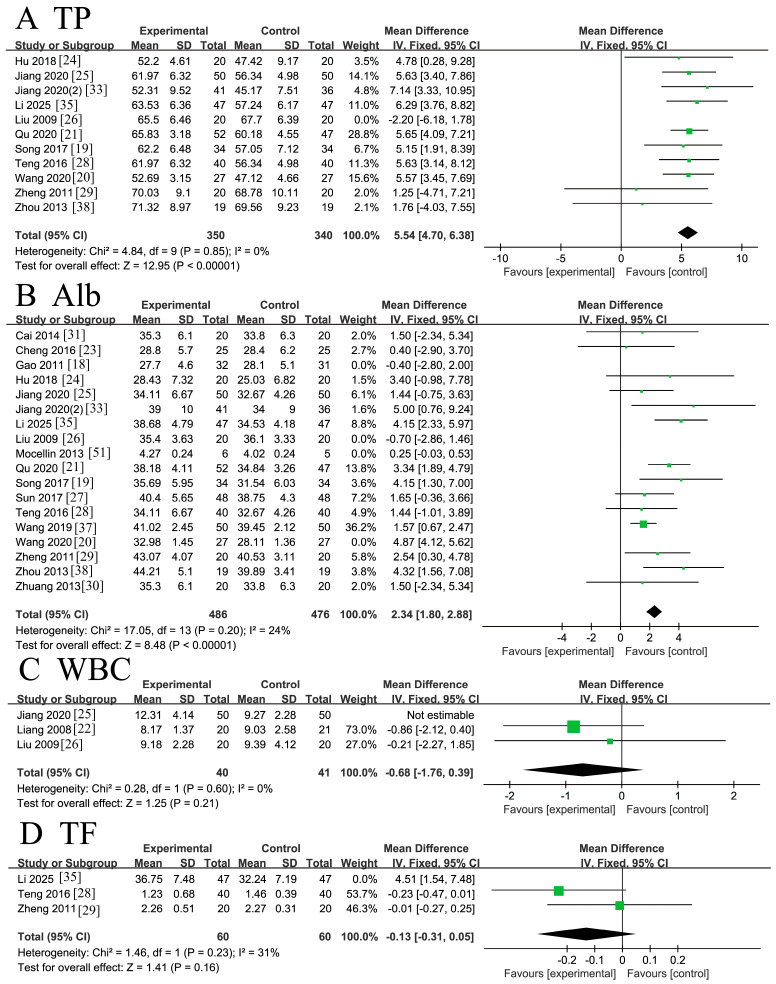
Meta-analysis of blood indicators of recovery after CRC surgery with ω-3 PUFAs. (**A**) TP: serum total protein; (**B**) Alb: serum albumin; (**C**) WBC: white blood cell; (**D**) TF: transcription factor. Note: Each square represents the effect estimate (mean difference) of an individual study, with the size of the square proportional to the study weight. Horizontal lines indicate the corresponding 95% confidence intervals (CIs). The vertical solid line represents the line of no effect (mean difference = 0). The black diamond represents the pooled effect estimate, with its width indicating the 95% CI. Effect estimates located to the left or right of the vertical line favor the experimental or control group, respectively.

Eleven RCTs [19,20,21,24,25,26,28,29,33,35,38] involving 730 patients reported data on serum total protein (TP). Initial assessment indicated significant heterogeneity (*I*^2^ = 47%, *p* = 0.04 < 0.1). Sensitivity analysis identified the study by Liu 2009 [26] as the primary source of heterogeneity. After its exclusion, the remaining 10 studies showed acceptable homogeneity (*I*^2^ = 0% < 50%, *p* = 0.85 > 0.1). A fixed-effects model meta-analysis of these 10 studies demonstrated that ω-3 PUFA supplementation significantly increased TP levels in CRC patients compared to controls [MD = 5.54, 95% CI (4.70, 6.38), Z = 12.95, *p* < 0.00001]. The forest plot is presented in Figure 4A.

Fourteen RCTs [18,20,21,23,24,25,26,27,28,29,33,36,38,50,51] involving 1130 patients reported serum albumin (Alb) levels. The study by Mocellin 2013 [51] was excluded due to a unit error that rendered its data incompatible. The initial analysis of the remaining studies revealed substantial heterogeneity (*I*^2^ =90% > 50%, Q-test *p* < 0.00001). Sensitivity analysis identified Gao 2011 [18], Liu 2009 [26], and Wang 2020 [20] as major contributors. After excluding these three studies, the remaining 10 trials exhibited acceptable homogeneity (*I*^2^ = 24% < 50%, *p* = 0.20 > 0.1). A fixed-effects model meta-analysis of these 10 studies indicated a statistically significant elevation in Alb levels following ω-3 PUFA supplementation [MD = 2.34, 95%CI (1.80, 2.88), Z = 8.48, *p* < 0.00001]. The forest plot is shown in Figure 4B.

Three RCTs [22,25,26] involving 81 patients reported white blood cell (WBC) counts. Significant heterogeneity was observed among these studies (*I*^2^ = 89% > 50%, Q-test *p* < 0.0001). Sensitivity analysis pinpointed Jiang 2020 [25] as the primary source. After its exclusion, the remaining two studies were homogeneous (*I*^2^ = 0% < 50%, *p* = 0.60 > 0.1). A fixed-effects model meta-analysis found no significant effect of ω-3 PUFA supplementation on WBC counts [MD = −0.68, 95% CI (−1.76, 0.39), Z = 1.25, *p* = 0.21 > 0.05]. The forest plot is presented 6.

Three RCTs [28,29,35] involving 214 patients reported transcription factor (TF) levels. The study by Li 2025 [35] was excluded a priori due to inconsistencies in the reported statistical units. The remaining studies demonstrated acceptable homogeneity (*I*^2^ = 31% < 50%, Q-test *p* = 0.23 > 0.1). Sensitivity analysis confirmed that the pooled estimate was robust and not unduly influenced by any single study. A fixed-effects model meta-analysis revealed no statistically significant effect of ω-3 PUFA supplementation on TF levels in CRC patients [MD = −0.13, 95% CI (−0.31, 0.05), Z = 1.41, *p* = 0.16]. The forest plot is presented in Figure 4D.

#### 3.4.2. Blood Lymphocyte Indicators

Ten RCTs [19,21,22,24,26,28,29,30,34,35] involving 642 patients reported the levels of CD3^+^ T cells (%). Significant heterogeneity was observed across these studies (*I*^2^ = 61% > 50%, Q-test *p* = 0.006 < 0.1). Sensitivity analysis identified Qu 2020 [21] as the major source of heterogeneity. After excluding this study, the remaining nine studies showed acceptable homogeneity (*I*^2^ = 0% < 50%, *p* = 0.59 > 0.1). Meta-analysis using a fixed-effects model yielded two main findings:

1. CD3^+^ T cell levels were significantly higher in patients receiving ω-3 PUFA supplementation compared with controls;
2. A greater postoperative increase in CD3^+^ T cell levels was observed in the ω-3 PUFAs group relative to the placebo group.

**Figure 5:**
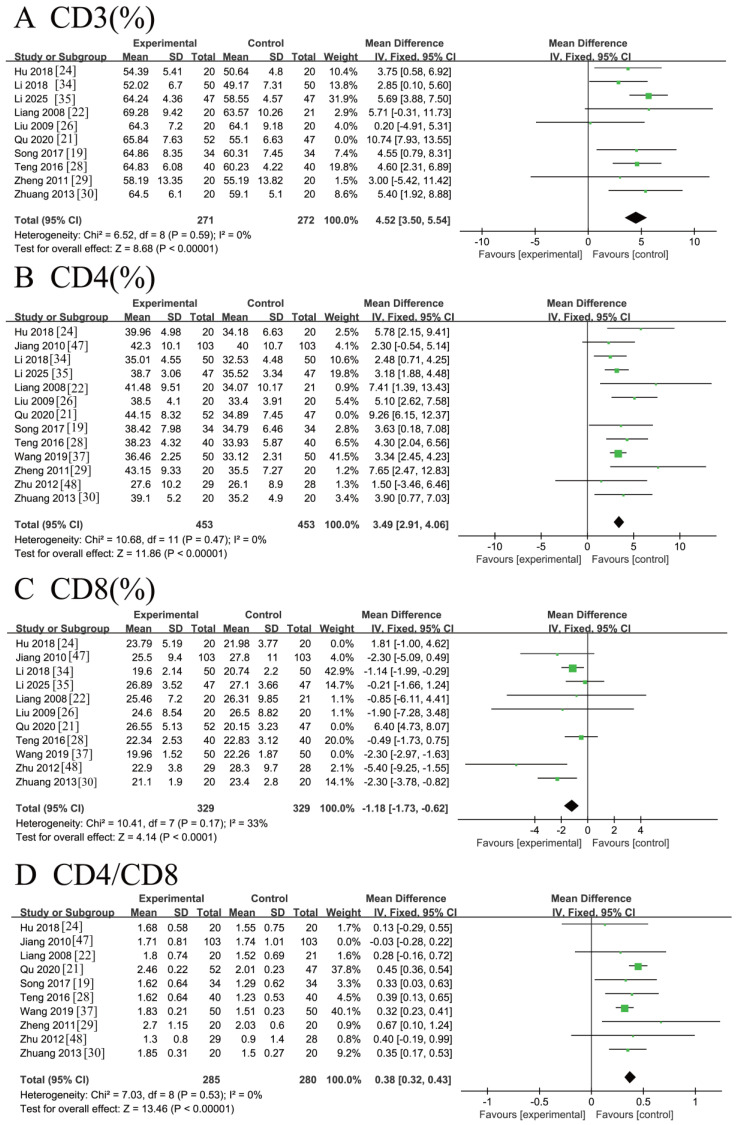
Meta-analysis of blood lymphocyte indicators of recovery after CRC surgery with ω-3 PUFAs intervention. (**A**) CD3 (%): CD3^+^ T cells (%) levels; (**B**) CD4 (%): CD4^+^ T cell (%) levels; (**C**) CD8 (%): CD8^+^ T cell (%) levels; (**D**) CD4/CD8: CD4^+^/CD8^+^ T cell ratio. Note: Each square represents the effect estimate (mean difference) of an individual study, with the size of the square proportional to the study weight. Horizontal lines indicate the corresponding 95% confidence intervals (CIs). The vertical solid line represents the line of no effect (mean difference = 0). The black diamond represents the pooled effect estimate, with its width indicating the 95% CI. Effect estimates located to the left or right of the vertical line favor the experimental or control group, respectively.

The pooled analysis demonstrated a statistically significant effect: [MD = 4.52, 95% CI (3.50, 5.54), Z = 8.68, *p* < 0.00001]. The corresponding forest plot is shown in Figure 5A.

Thirteen RCTs [19,21,22,24,26,28,29,30,34,35] involving 1005 patients reported CD4^+^ T cell (%) levels. Heterogeneity analysis showed borderline significance (*I*^2^ = 49%; Q-test *p* = 0.02), indicating statistically significant heterogeneity among the studies. Sensitivity analysis identified Qu 2020 [21] as the main source of heterogeneity. After its exclusion, the remaining twelve studies exhibited acceptable homogeneity (*I*^2^ = 0%, *p* = 0.47). A fixed-effects meta-analysis of these homogeneous studies indicated a statistically significant increase in CD4^+^ T cell levels among CRC patients receiving ω-3 PUFA supplementation compared with placebo controls: [MD = 3.49, 95% CI (2.91, 4.06), Z = 11.86, *p* < 0.00001]. The corresponding forest plot is shown in Figure 5B.

Eleven RCTs [21,22,24,26,28,30,34,35,37,47,48] involving 897 patients reported CD8^+^ T cell (%) levels. Significant heterogeneity was observed (*I*^2^ = 90% > 50%, Q-test *p* < 0.00001). A sensitivity analysis was performed to address this, leading to the exclusion of three studies (Qu 2020 [21], Hu 2018 [24], Wang 2019 [37]) identified as major contributors. The meta-analysis of the remaining eight homogeneous studies (*I*^2^ = 33%, *p* = 0.17 > 0.1) yielded a robust estimate, indicating a significant decrease in CD8^+^ T cell levels with ω-3 PUFA supplementation [MD = −1.18, 95% CI (−1.73, −0.62), Z = 4.14, *p* < 0.0001]. The forest plot is presented in Figure 5C.

Ten RCTs [19,21,22,24,28,29,30,37,47,48] involving 771 patients reported the CD4^+^/CD8^+^ T cell ratio. Initial assessment revealed moderate heterogeneity (*I*^2^ = 46% < 50%, but Q-test *p* = 0.05 < 0.1). Sensitivity analysis identified the study by Jiang 2010 [47] as the primary source of heterogeneity. After its exclusion, the remaining nine studies demonstrated acceptable homogeneity (*I*^2^ = 0% < 50%, *p* = 0.53 > 0.1). A fixed-effects model meta-analysis of these homogeneous studies indicated a statistically significant increase in the CD4^+^/CD8^+^ ratio following ω-3 PUFA supplementation compared to placebo controls [MD = 0.38, 95%CI (0.32, 0.43), Z = 13.46, *p* < 0.00001]. The forest plot is presented in Figure 5D.

#### 3.4.3. Inflammatory Indicators

**Figure 6:**
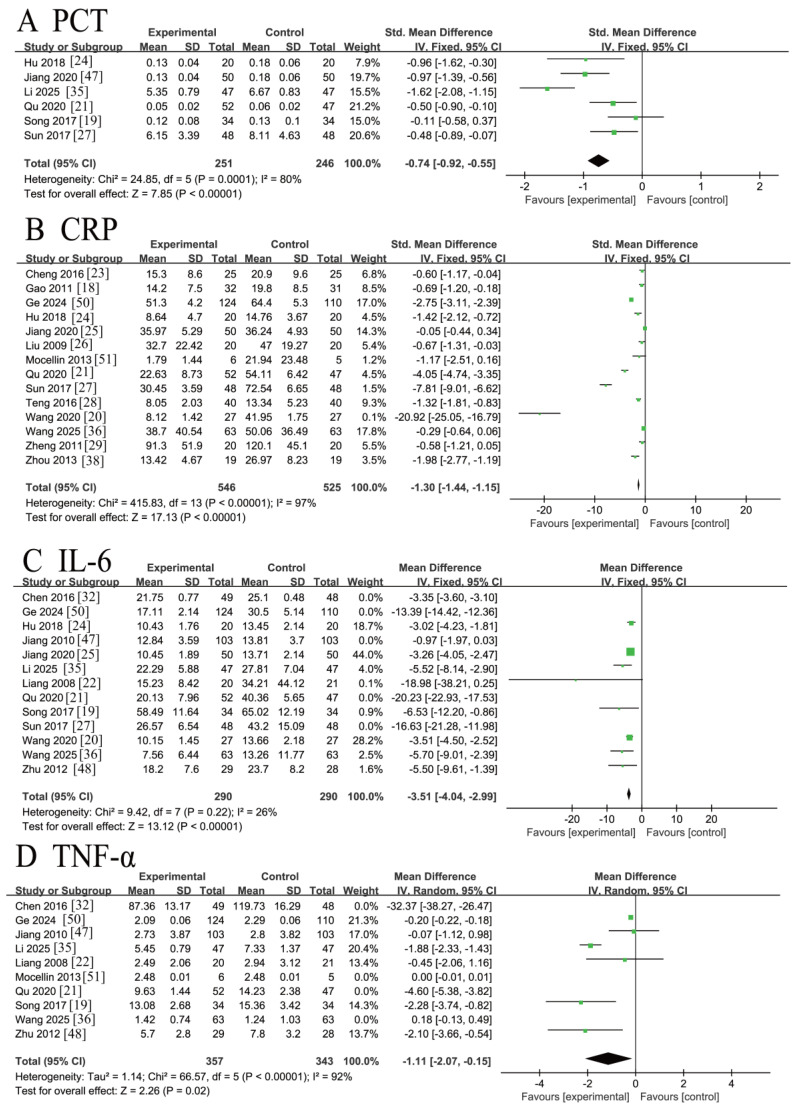
Meta-analysis of inflammatory indicators of recovery after CRC surgery with ω-3 PUFAs. (**A**) PCT: procalcitonin; (**B**) CRP: C-reactive protein; (**C**) IL-6: interleukin-6; (**D**) TNF-α: factor-α. Note: Each square represents the effect estimate (mean difference) of an individual study, with the size of the square proportional to the study weight. Horizontal lines indicate the corresponding 95% confidence intervals (CIs). The vertical solid line represents the line of no effect (mean difference = 0). The black diamond represents the pooled effect estimate, with its width indicating the 95% CI. Effect estimates located to the left or right of the vertical line favor the experimental or control group, respectively.

Six RCTs [19,21,24,25,27,35] involving 497 patients reported procalcitonin (PCT) levels. The initial meta-analysis revealed substantial heterogeneity (*I*^2^ = 80% > 50%, Q-test *p* = 0.0001 < 0.1). Sensitivity analysis identified the studies by Li 2025 [35] and Song 2017 [19] as the primary sources of heterogeneity. After excluding these two studies, the remaining four studies demonstrated acceptable homogeneity (*I*^2^ = 31% < 50%, *p* = 0.23 > 0.1). A fixed-effects model meta-analysis of these four studies indicated that ω-3 PUFA supplementation significantly reduced PCT levels compared to placebo [MD = −0.68, 95%CI (−0.90, −0.46), Z = 6.01, *p* < 0.00001]. The forest plot is presented in Figure 6A.

Fourteen RCTs [18,21,23,24,25,26,27,28,29,36,37,38,50,51] involving 1071 patients reported C-reactive protein (CRP) levels. Extreme heterogeneity was observed across the included studies (*I*^2^ = 97% > 50%, Q-test *p* < 0.00001). Sensitivity analysis indicated that this substantial heterogeneity originated from multiple sources, potentially including variations in ω-3 PUFAs dosage regimens, differences in CRP assay methodologies, and timing of biomarker assessment. Given the irreducible nature of the heterogeneity, a random-effects model was employed for the meta-analysis. The pooled results demonstrated a statistically significant reduction in CRP levels among CRC patients receiving ω-3 PUFA supplementation compared to placebo controls [MD = −16.90, 95% CI (−28.36, −5.43), Z = 2.89, *p* = 0.004 < 0.05]. The corresponding forest plot is presented in Figure 6B.

Thirteen RCTs [19,21,22,24,25,27,32,35,37,47,48,50] involving 1312 patients reported interleukin-6 (IL-6) levels. The initial analysis revealed substantial heterogeneity (*I*^2^ = 98% > 50%, Q-test *p* < 0.00001). Sensitivity analysis identified five studies (Jiang 2010 [47], Qu 2020 [21], Sun 2017 [27], Chen 2016 [32], Ge 2024 [50]) as the primary sources of heterogeneity. After their exclusion, the remaining eight studies demonstrated acceptable homogeneity (*I*^2^ = 26% < 50%, *p* = 0.22 > 0.1). A fixed-effects model meta-analysis of these eight studies demonstrated a statistically significant reduction in IL-6 levels following ω-3 PUFA supplementation compared to placebo controls [MD = −3.51, 95% CI (−4.04, −2.99), Z = 13.12, *p* < 0.00001]. The forest plot is presented in Figure 6C.

Ten RCTs [19,21,22,32,35,36,47,48,50,51] involving 1033 patients reported tumor necrosis factor-α (TNF-α) levels. Significant heterogeneity was observed among the studies (*I*^2^ = 99% > 50%, Q-test *p*< 0.00001). Sensitivity analysis identified four studies (Chen 2016 [32], Qu 2020 [21], Mocellin 2013 [51], Wang 2025 [36]) as primary sources of heterogeneity. Despite their exclusion, substantial heterogeneity persisted in the remaining six studies (*I^2^* = 92%, *p* < 0.00001). Consequently, a random-effects model was employed for the meta-analysis. The pooled results demonstrated a statistically significant reduction in TNF-α levels in CRC patients receiving ω-3 PUFA supplementation compared to the placebo group [MD = −1.11, 95%CI (−2.07, −0.15), Z = 2.26, *p* = 0.02 < 0.05]. The forest plot for this analysis is presented in Figure 6D.

#### 3.4.4. Immune Indicators

**Figure 7:**
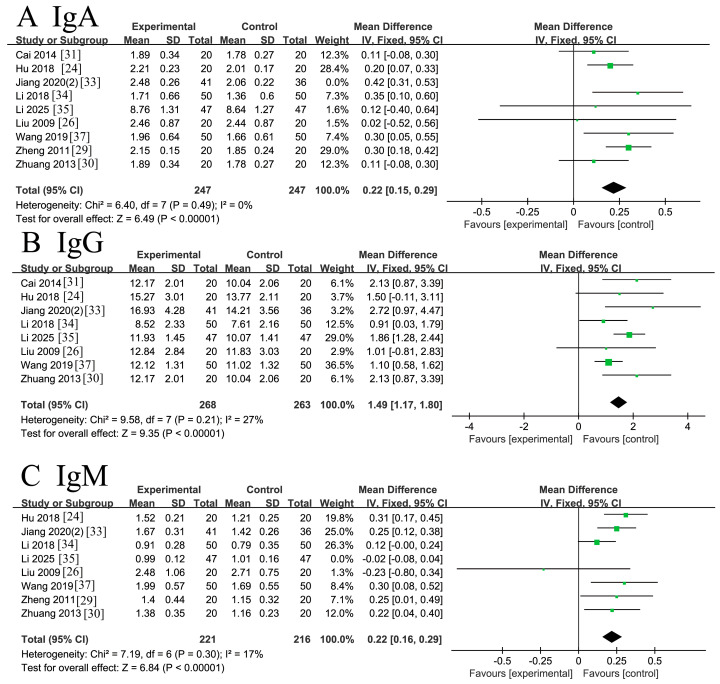
Meta-analysis of immune indicators of recovery after CRC surgery with ω-3 PUFAs. (**A**) IgA: Immunoglobulin A; (**B**) IgG: Immunoglobulin G; (**C**) IgC: Immunoglobulin C. Note: Each square represents the effect estimate (mean difference) of an individual study, with the size of the square proportional to the study weight. Horizontal lines indicate the corresponding 95% confidence intervals (CIs). The vertical solid line represents the line of no effect (mean difference = 0). The black diamond represents the pooled effect estimate, with its width indicating the 95% CI. Effect estimates located to the left or right of the vertical line favor the experimental or control group, respectively.

Nine RCTs [24,26,29,30,31,33,34,35,37] involving 494 patients reported immunoglobulin A (IgA) levels. The initial analysis revealed moderate heterogeneity (*I*^2^ = 50%, *p* = 0.04< 0.1). Sensitivity analysis identified the study by Jiang 2020(2) [33] as the primary source of heterogeneity. After its exclusion, the remaining eight studies demonstrated low heterogeneity (*I*^2^ = 0%, *p* = 0.49). A fixed-effects model meta-analysis of these eight studies indicated a statistically significant increase in IgA levels following ω-3 PUFA supplementation compared to placebo controls [MD = 0.22, 95% CI (0.15, 0.29), Z = 6.49, *p* < 0.00001]. The forest plot is shown in Figure 7A.

Eight RCTs [24,26,30,31,33,34,35,37] involving 531 patients reported immunoglobulin G (IgG) levels. The analysis demonstrated low heterogeneity (*I*^2^ = 27%, *p* = 0.21 > 0.1). Sensitivity analysis confirmed the robustness of the results, as no single study substantially influenced the pooled estimates. A fixed-effects model meta-analysis revealed a statistically significant increase in IgG levels in the ω-3 PUFAs group compared to the placebo group [MD = 1.49, 95% CI (1.17, 1.80), Z = 9.35, *p* < 0.00001]. The forest plot is presented in Figure 7B.

Eight RCTs [24,26,29,30,33,34,35,37] involving 437 patients reported immunoglobulin M (IgM) levels. The initial analysis revealed substantial heterogeneity (*I*^2^ = 82%, Q-test *p* < 0.00001). Sensitivity analysis identified the study by Li 2025 [35] as the primary source of heterogeneity. After its exclusion, the remaining seven studies demonstrated low heterogeneity (*I*^2^ = 17% < 50%, *p* = 0.30 > 0.1). A fixed-effects model meta-analysis of these seven studies indicated a statistically significant increase in IgM levels following ω-3 PUFA supplementation compared to controls [MD = 0.22, 95% CI (0.16, 0.29), Z = 6.84, *p* < 0.00001]. The forest plot is shown in Figure 7C.

#### 3.4.5. Quality-of-Life Indicators

**Figure 8:**
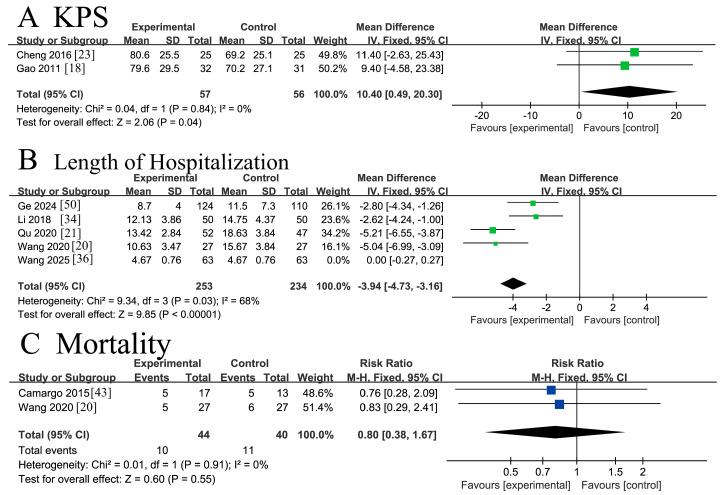
Meta-analysis of quality-of-life indicators of recovery after CRC surgery with ω-3 PUFAs. (**A**) KPS: Karnofsky Performance Status; (**B**) Length of Hospitalization: the postoperative length of hospitalization; (**C**) Mortality: postoperative mortality. Note: Each square represents the effect estimate (mean difference) of an individual study, with the size of the square proportional to the study weight. Horizontal lines indicate the corresponding 95% confidence intervals (CIs). The vertical solid line represents the line of no effect (mean difference = 0). The black diamond represents the pooled effect estimate, with its width indicating the 95% CI. Effect estimates located to the left or right of the vertical line favor the experimental or control group, respectively. Each blue square represents the effect estimate (risk ratio, RR) of an individual study, with the size of the square proportional to the study weight. The horizontal line through each square indicates the corresponding 95% confidence interval (CI). The vertical solid line represents the line of no effect (RR = 1). The black diamond represents the pooled effect estimate, with its width indicating the 95% CI. Effect estimates located to the left or right of the vertical line favor the experimental or control group, respectively.

Two RCTs [18,23] involving 113 patients reported the Karnofsky Performance Status (KPS). The analysis demonstrated low heterogeneity (*I*^2^ = 0%, *p* = 0.84 > 0.1), and sensitivity analysis confirmed the robustness of the pooled estimates. A fixed-effects model meta-analysis showed a statistically significant improvement in KPS scores among CRC patients receiving ω-3 PUFA supplementation compared to placebo controls [MD = 10.40, 95% CI (0.49, 20.30), Z = 2.06, *p* = 0.04 < 0.05]. The considerable confidence interval width suggests a potential benefit, albeit with imprecision in the effect size estimate. The forest plot is presented in Figure 8A.

Five RCTs [20,21,34,36,50] involving 613 patients reported the postoperative length of hospitalization. Initial analysis revealed substantial heterogeneity among the studies (*I*^2^ = 96% > 50%, Q-test *p* < 0.00001). Sensitivity analysis identified the study by Wang 2025 [36] as a major source of heterogeneity. After its exclusion, significant heterogeneity persisted among the remaining four studies (*I*^2^ = 68% > 50%, Q-test *p* =0.03 < 0.1). Further sensitivity analyses confirmed that all four studies contributed substantially to the residual heterogeneity. Given this irreducible heterogeneity, a random-effects model was applied. The meta-analysis demonstrated a statistically significant reduction in hospitalization duration for CRC patients receiving ω-3 PUFA supplementation compared to the placebo group [MD = −3.91, 95% CI (−5.31, −2.50), Z = 5.45, *p* < 0.00001]. The forest plot is shown in Figure 8B.

Two RCTs [20,43] involving 84 patients reported postoperative mortality. No heterogeneity was observed across the studies (*I*^2^ = 0%, *p* = 0.91 > 0.1). Sensitivity analysis confirmed the robustness of the pooled estimate, as no single trial exerted a significant influence on the results. A fixed-effects model meta-analysis showed no statistically significant effect of ω-3 PUFA supplementation on postoperative mortality in CRC patients [RR = 0.80, 95% CI (0.38, 1.67), Z = 0.60, *p* = 0.55 > 0.05]. The forest plot is presented in Figure 8C.

#### 3.4.6. Complications Comparison Results

**Figure 9:**
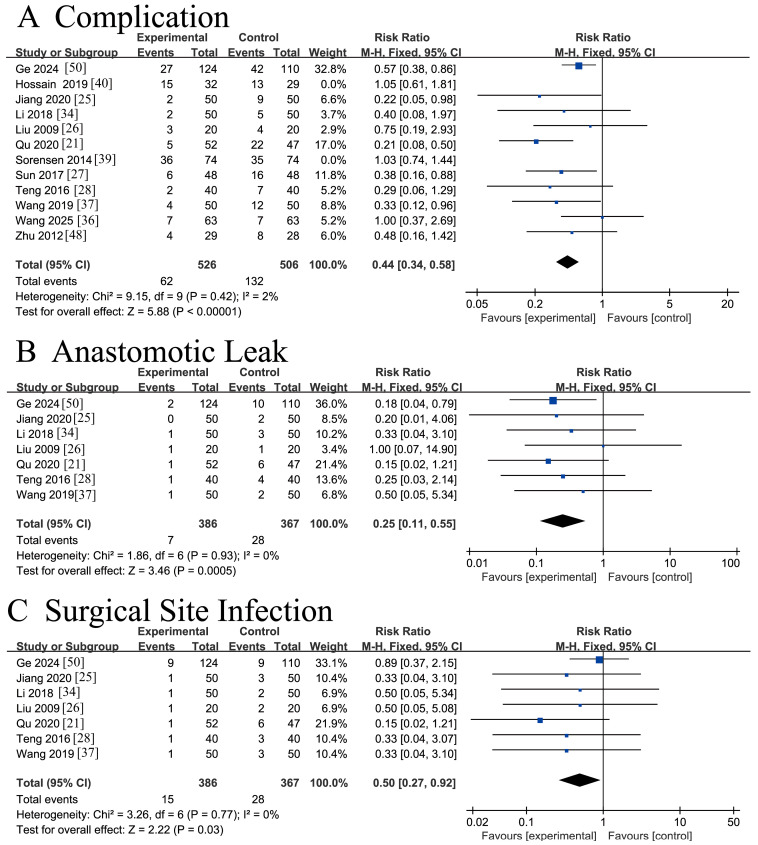
Meta-analysis of complications comparison of recovery after CRC surgery with ω-3 PUFAs. (**A**) Complication: postoperative complications; (**B**) Anastomotic Leak; (**C**) IgC: Surgical Site Infection. Note: Each blue square represents the effect estimate (risk ratio, RR) of an individual study, with the size of the square proportional to the study weight. The horizontal line through each square indicates the corresponding 95% confidence interval (CI). The vertical solid line represents the line of no effect (RR = 1). The black diamond represents the pooled effect estimate, with its width indicating the 95% CI. Effect estimates located to the left or right of the vertical line favor the experimental or control group, respectively.

Twelve RCTs [21,25,26,27,28,34,36,37,39,40,48,50] involving 1241 patients reported postoperative complication rates. Significant heterogeneity was observed across the studies (*I*^2^ = 58% > 50%, Q-test *p* = 0.006 < 0.1). Sensitivity analysis identified the studies by Hossain 2019 [40] and Sorensen 2014 [39] as the primary sources of heterogeneity. After their exclusion, the remaining ten studies demonstrated low heterogeneity (*I*^2^ = 2% < 50%, *p* = 0.42 > 0.1). A fixed-effects model meta-analysis of these ten studies indicated a statistically significant reduction in postoperative complications among CRC patients receiving ω-3 PUFA supplementation compared to placebo controls [RR = 0.44, 95% CI (0.34, 0.58), Z = 5.88, *p* < 0.00001]. The forest plot is presented in Figure 9A.

Seven RCTs [21,25,26,28,34,37,50] involving 753 patients reported the incidence of postoperative anastomotic leak. No heterogeneity was observed among the studies (*I*^2^ = 0% < 50%, Q-test *p* = 0.93 > 0.1). Sensitivity analysis confirmed that the pooled results were not substantially influenced by any single trial. A fixed-effects model meta-analysis demonstrated a statistically significant reduction in anastomotic leak among CRC patients receiving ω-3 PUFA supplementation compared with placebo controls [RR = 0.25, 95%CI (0.11, 0.55), Z = 3.46, *p* = 0.005 < 0.05]. The forest plot is presented in Figure 9B.

Seven RCTs [21,25,26,28,34,37,50] involving 753 patients reported the incidence of surgical site infection (SSI). No heterogeneity was observed among the included studies (*I*^2^ = 0% < 50%, Q-test *p* = 0.77 > 0.1). Sensitivity analysis confirmed that no single trial substantially influenced the pooled results. A fixed-effects model meta-analysis revealed a statistically significant reduction in SSI among CRC patients receiving ω-3 PUFA supplementation compared with placebo controls [RR = 0.50, 95%CI (0.27, 0.92), Z = 2.22, *p* = 0.03 < 0.05]. The corresponding forest plot is presented in Figure 9C.

#### 3.4.7. Other Indicators

**Figure 10:**
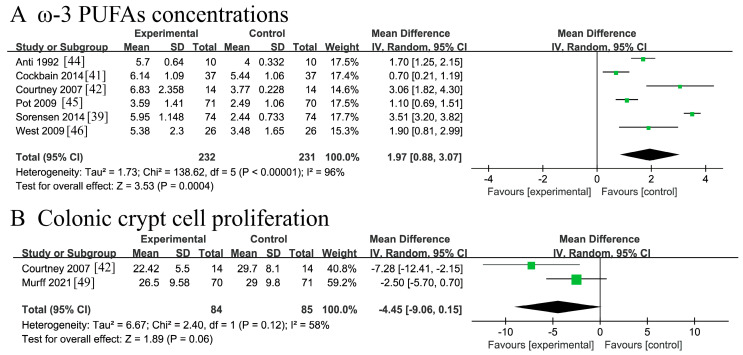
Meta-analysis of other indicators of recovery after CRC surgery with ω-3 PUFAs. (**A**) ω-3 PUFAs concentrations; (**B**) colonic crypt cell proliferation. Note: Each square represents the effect estimate (mean difference) of an individual study, with the size of the square proportional to the study weight. Horizontal lines indicate the corresponding 95% confidence intervals (CIs). The vertical solid line represents the line of no effect (mean difference = 0). The black diamond represents the pooled effect estimate, with its width indicating the 95% CI. Effect estimates located to the left or right of the vertical line favor the experimental or control group, respectively.

Six RCTs [39,41,42,44,45,46] involving 463 patients reported ω-3 PUFAs concentrations. Substantial heterogeneity was observed across studies (*I*^2^ = 96% > 50%, Q-test *p* < 0.00001), potentially attributable to variations in: (1) intervention dosage, (2) analytical methodologies, (3) timing of sample collection, and (4) biological matrices (e.g., plasma versus rectal mucosa). A random-effects meta-analysis demonstrated a statistically significant increase in ω-3 PUFAs concentrations in CRC patients receiving supplementation compared with placebo controls [MD =1.97, 95%CI (0.88, 3.07), Z = 3.53, *p* = 0.0004 < 0.05]. The forest plot is presented in Figure 10A.

Two RCTs [42,49] involving 169 patients reported colonic crypt cell proliferation indices. Heterogeneity analysis indicated moderate heterogeneity between the studies (*I*^2^ = 58% > 50%). As both trials contributed to the observed heterogeneity, a random-effects model was employed for the meta-analysis. The pooled results demonstrated a strong trend toward reduced crypt cell proliferation in CRC patients receiving ω-3 PUFA supplementation, although this association did not reach conventional statistical significance [MD = −4.45, 95% CI (−9.06, 0.15), Z = 1.89, *p* = 0.06 > 0.05]. The forest plot is presented in Figure 10B.

### 3.5. Publication Bias Test

**Figure 11:**
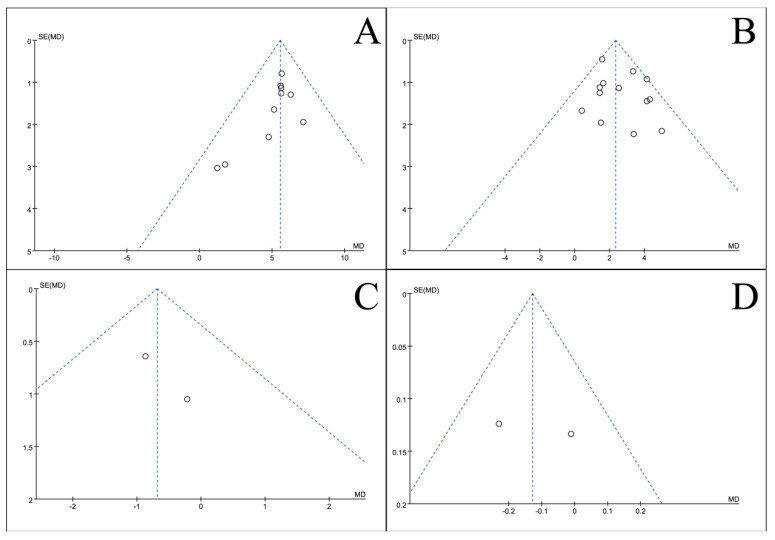
Inverted funnel plot of blood indicators of recovery after CRC surgery with ω-3 PUFAs. (**A**) TP; (**B**) Alb; (**C**) WBC; (**D**) TF. Note: Each circle represents an individual study. The vertical dotted line indicates the pooled effect estimate, and the diagonal dotted lines represent the pseudo 95% confidence limits. Funnel plots are used to visually assess potential publication bias or small-study effects.

**Figure 12:**
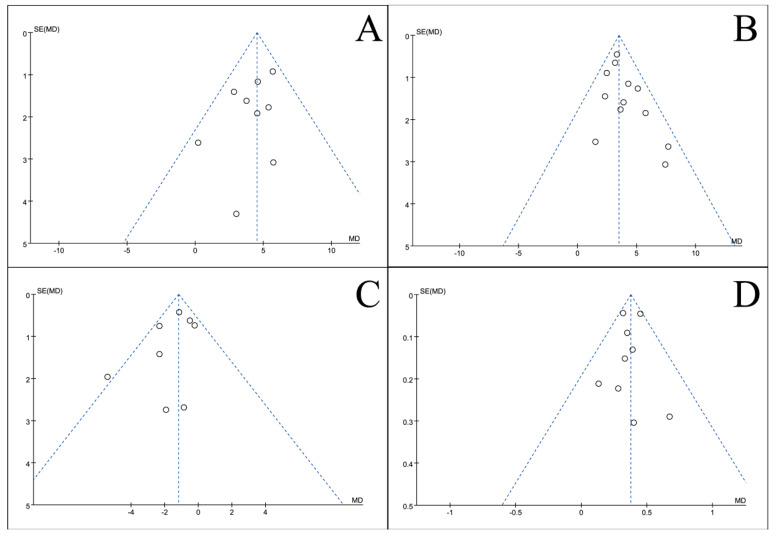
Inverted funnel plot of blood lymphocyte indicators of recovery after CRC surgery with ω-3 PUFAs. (**A**) CD3^+^T cell (%); (**B**) CD4^+^T cell (%); (**C**) CD8^+^T cell (%); (**D**) CD4^+^/CD8^+^T. Note: Each circle represents an individual study. The vertical dotted line indicates the pooled effect estimate, and the diagonal dotted lines represent the pseudo 95% confidence limits. Funnel plots are used to visually assess potential publication bias or small-study effects.

**Figure 13:**
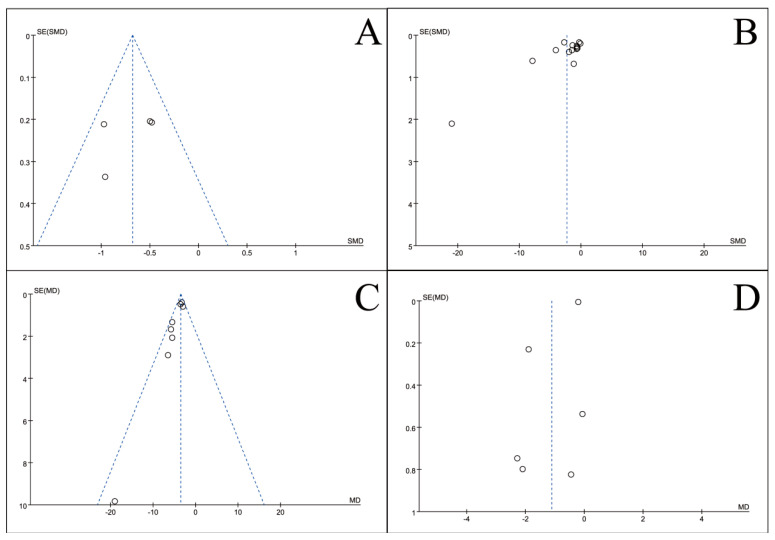
Inverted funnel plot of inflammatory indicators of recovery after CRC surgery with ω-3 PUFAs. (**A**) PCT; (**B**) CRP; (**C**) IL-6; (**D**) TNF-α. Note: Each circle represents an individual study. The vertical dotted line indicates the pooled effect estimate, and the diagonal dotted lines represent the pseudo 95% confidence limits. Funnel plots are used to visually assess potential publication bias or small-study effects.

**Figure 14:**
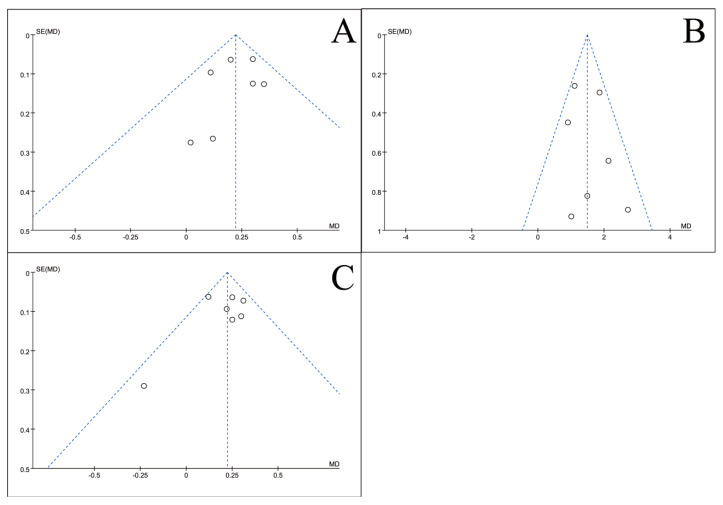
Inverted funnel plot of inflammatory indicators of recovery after CRC surgery with ω-3 PUFAs. (**A**) IgA; (**B**) IgG; (**C**) IgM. Note: Each circle represents an individual study. The vertical dotted line indicates the pooled effect estimate, and the diagonal dotted lines represent the pseudo 95% confidence limits. Funnel plots are used to visually assess potential publication bias or small-study effects.

**Figure 15:**
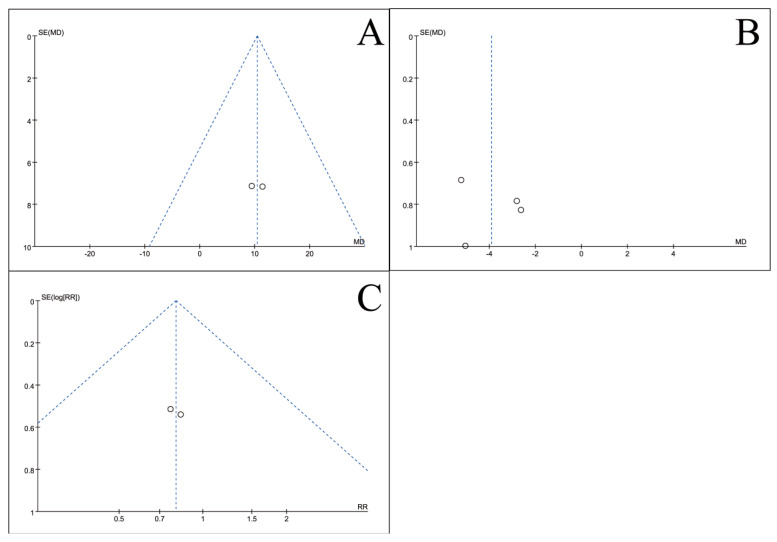
Inverted funnel plot of quality-of-life indicators of recovery after CRC surgery with ω-3 PUFAs. (**A**) KPS; (**B**) Length of hospitalization; (**C**) Mortality rate. Note: Each circle represents an individual study. The vertical dotted line indicates the pooled effect estimate, and the diagonal dotted lines represent the pseudo 95% confidence limits. Funnel plots are used to visually assess potential publication bias or small-study effects.

**Figure 16:**
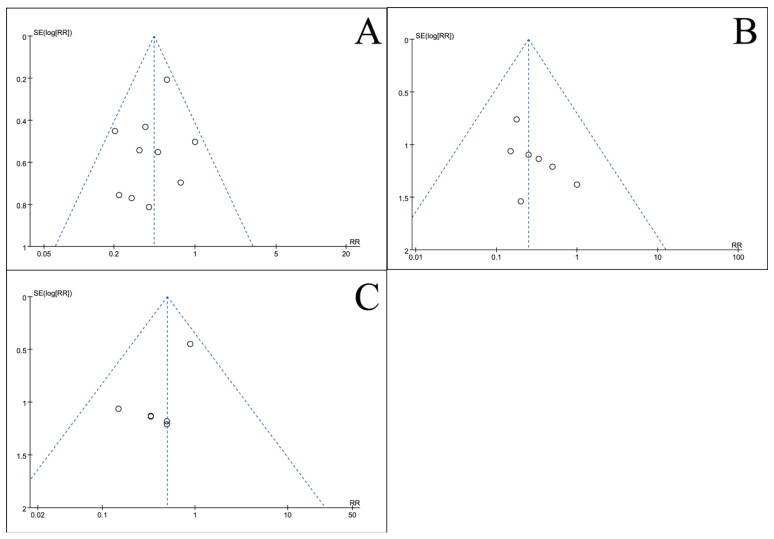
Inverted funnel plot of complications comparison of recovery after CRC surgery with ω-3 PUFAs. (**A**) Complications; (**B**) Anastomotic Leak; (**C**) Incision infection. Note: Each circle represents an individual study. The vertical dotted line indicates the pooled effect estimate, and the diagonal dotted lines represent the pseudo 95% confidence limits. Funnel plots are used to visually assess potential publication bias or small-study effects.

**Figure 17:**
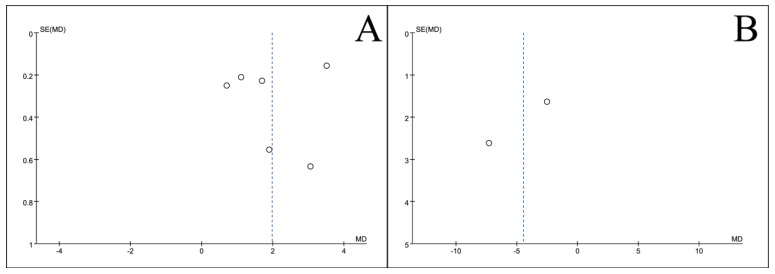
Inverted funnel plot of other indicators of recovery after CRC surgery with ω-3 PUFAs. (**A**) CONC of ω-3 PUFAs; (**B**) Crypt cell proliferation. Note: Each circle represents an individual study. The vertical dotted line indicates the pooled effect estimate, and the diagonal dotted lines represent the pseudo 95% confidence limits. Funnel plots are used to visually assess potential publication bias or small-study effects.

To assess potential publication bias, inverted funnel plots were constructed for all outcome measures included in the meta-analyses. Visual inspection showed broad symmetry across all funnel plots (Figure 11, Figure 12, Figure 13, Figure 14, Figure 15, Figure 16 and Figure 17), suggesting little evidence of publication bias.

### 3.6. Results of the Optimal Intake Dose Study

**Figure 18:**
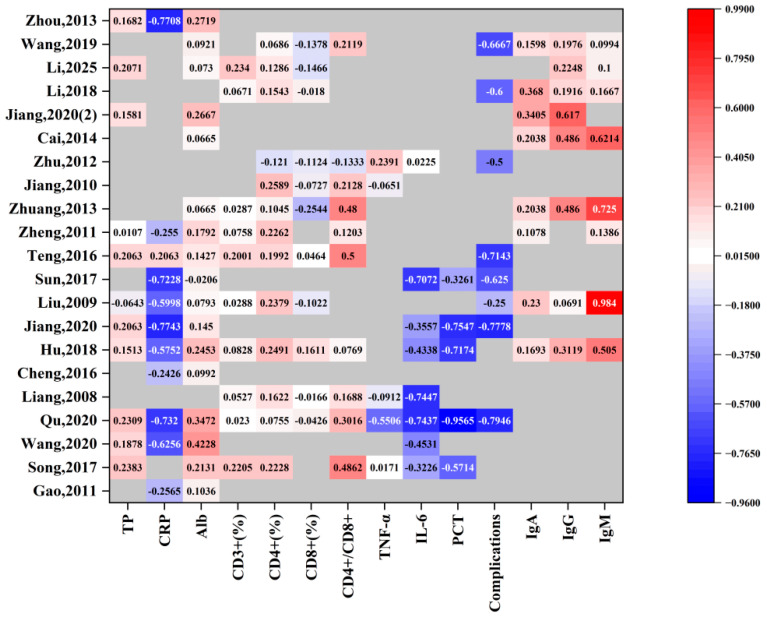
Heatmap of rate of change in outcome indicators for several RCTs. Note: The color scale represents the direction and magnitude of changes in each index following ω-3 PUFA supplementation. Red indicates an increase in the index, with darker red representing a greater magnitude of increase, whereas blue indicates a decrease in the index, with darker blue representing a greater magnitude of decrease. Lighter colors (approaching white) indicate minimal or no change. The numerical values shown in each cell correspond to the magnitude of change.

Analysis of data from 21 RCTs involving Chinese CRC patients revealed considerable heterogeneity in the effects of different daily ω-3 PUFAs doses and treatment durations on tumor markers. As illustrated in Figure 18, the regimen from Qu 2020—comprising one week of preoperative and one week of postoperative intervention at a daily dose of 0.31 g/kg—was associated with significant improvements across multiple biomarkers. Other studies, including Jiang 2010 and Teng 2016, also demonstrated beneficial effects on specific outcome measures. Synthesizing these findings, a daily ω-3 PUFAs intake ranging from 0.16 to 0.31 g/kg is proposed as an optimal dose window for enhancing postoperative recovery in CRC patients.

## 4. Discussion

This meta-analysis included 34 RCTs [18,19,20,21,22,23,24,25,26,27,28,29,30,31,32,33,34,35,36,37,38,39,40,41,42,43,44,45,46,47,48,49,50,51] involving 2889 CRC patients and provides robust evidence that perioperative ω-3 PUFA supplementation significantly improves postoperative recovery. The intervention yielded significant benefits across multiple domains, including the restoration of blood parameters (e.g., total protein), attenuation of systemic inflammation (e.g., C-reactive protein), and a notable reduction in complication rates. Furthermore, the general symmetry observed in the inverted funnel plots (Figure 11, Figure 12, Figure 13, Figure 14, Figure 15, Figure 16 and Figure 17) suggests a low risk of substantial publication bias. These findings collectively support the integration of ω-3 PUFAs into the perioperative nutritional management of CRC patients.

Key strengths of this study include: (1) the exclusive inclusion of RCTs enhances the methodological quality, with recent evidence published in the past five years; (2) the comprehensive assessment of clinically relevant endpoints, providing a holistic view of postoperative recovery; and (3) this is the first meta-analysis to systematically assess the efficacy and safety of ω-3 PUFA supplementation, specifically focusing on postoperative complications in CRC patients. However, several limitations should be considered: (1) potential language bias from the exclusion of non-English studies; (2) unreported or incomplete reporting of attrition rates across trials; (3) clinical heterogeneity stemming from variations in disease severity, therapeutic regimens, and regional or nutritional disparities; and (4) This study only investigated the effects of omega-3 PUFA supplementation on postoperative recovery in patients with CRC, but did not distinguish between the effects of chronic (long-term) supplementation and acute (short-term) supplementation on postoperative recovery.

CRC, a malignancy of increasing global incidence, is frequently complicated by malnutrition and cachexia [52], This metabolic derangement is multifactorial, arising from tumor-induced reprogramming such as the Warburg effect [53], inflammatory cachexia mediated by cytokines (e.g., TNF-α, CRP, IL-6) that promote lipolysis and muscle proteolysis [54], and psycho-neuroendocrine disturbances associated with nutritional decline [55,56]. The imperative for perioperative nutritional optimization has thus brought ω-3 PUFAs to the fore, given their dual capacity to ameliorate nutritional status and exert immunomodulatory effects [57]. Our meta-analysis of 19 RCTs assessing postoperative blood parameters confirmed that ω-3 PUFAs significantly elevate total protein and Alb levels. The latter is of particular prognostic importance, as delayed Alb recovery postoperatively is associated with an elevated recurrence risk [58]. Furthermore, CRC pathogenesis involves profound immune dysregulation [59]. Tumor cells subvert immunity by suppressing IFN-γ in CD4^+^ T cells via Wnt signaling while promoting IL-17a production, thereby impairing antitumor surveillance [60]. Concurrently, chronic inflammation in the tumor microenvironment drives CD8^+^ T cell exhaustion, representing a major barrier to effective immunotherapy [61]. The CD4^+^/CD8^+^ ratio holds prognostic relevance, showing positive associations with optimized chemotherapeutic responses [62]. Our meta-analysis of 13 RCTs evaluating four immunologic endpoints showed that ω-3 PUFA supplementation significantly improved postoperative peripheral lymphocyte profiles, including levels of CD3^+^ T cells, CD4^+^ T cells, CD8^+^ T cells, and the CD4^+^/CD8^+^ ratio. These findings substantiate the immunorestorative potential of ω-3 PUFAs in counteracting surgery-induced immunosuppression, thereby contributing to improved therapeutic outcomes in CRC.

The initiation and progression of CRC are closely associated with a chronic inflammatory intestinal microenvironment, largely orchestrated by mediators such as CRP and IL-6 [63]. Clinical studies consistently report elevated circulating levels of TNF-α and IL-6 in CRC patients [64]. These cytokines contribute to tumorigenesis through multiple mechanisms; for example, under the persistent inflammatory stress of the tumor microenvironment, TNF-α shifts from its physiological pro-apoptotic role to a pathological one, promoting tumor progression via NF-κB-mediated activation of pro-metastatic genes [65]. Our meta-analysis, which synthesized data from 20 RCTs, confirms that ω-3 PUFA supplementation effectively counteracts this process, leading to significant reductions in PCT, CRP, IL-6, and TNF-α levels. This suppression of systemic inflammation highlights the pleiotropic anti-inflammatory properties of ω-3 PUFAs and confirms their role in optimizing postoperative recovery and overall CRC outcomes by targeting critical oncogenic pathways [66].

The effects of ω-3 PUFAs on humoral immunity in CRC patients are complex and multifaceted. Our meta-analysis, which synthesized data from 9 RCTs, demonstrated a consistent increase in serum levels of IgA, IgM, and IgG following ω-3 PUFA supplementation. While immunoglobulins are fundamental to immune competence—e.g., secretory IgA (sIgA) providing frontline mucosal defense [67] and IgG plays a central role in systemic humoral immunity [68]—their elevation in cancer patients requires cautious interpretation. This increase could represent an enhanced humoral response against tumor antigens. Conversely, it may also indicate immune dysregulation, as chronic antigenic stimulation from the tumor can lead to a compromised, dysregulated B-cell response [69]. Consequently, the immunomodulatory effect of ω-3 PUFAs on immunoglobulins, as observed here, is not inherently synonymous with improved clinical recovery. Its net effect on postoperative outcomes remains to be fully elucidated, highlighting a critical area for future mechanistic and clinical research.

Our meta-analysis of 8 RCTs demonstrates that ω-3 PUFA supplementation significantly improves key patient-centered outcomes in CRC patients, specifically by enhancing functional status as assessed by the KPS and reducing hospitalization length. The improvement in KPS is of particular clinical relevance, as it is an established indicator for better tolerance to chemotherapy and improved overall quality of life. KPS score greater than 50, it can be considered that the intervention measures to improve the tumor are considerable [70]. The absence of a significant mortality benefit may be attributable to the meta-analysis being underpowered for this endpoint. Consequently, while ω-3 PUFAs show promise in optimizing the efficiency and quality of recovery, their effect on long-term survival remains an open question that must be addressed in future large-scale, prospective studies.

Anastomotic leakage and surgical site infections (SSIs) are major drivers of postoperative morbidity and mortality following CRC surgery [71]. The consequences of these complications are particularly severe in resource-limited settings, where a prospective study found 30-day mortality rates to be four times higher than in high-income countries, despite comparable complication rates [72]. This finding underscores an urgent need for accessible strategies to improve surgical safety. Our meta-analysis of 12 RCTs indicates that ω-3 PUFA supplementation can reduce the incidence of postoperative complications, positioning it as a pragmatic intervention to enhance patient outcomes globally. The effective dosage was identified to be between 0.16 and 0.31 g/kg·day. Given the current absence of standardized ω-3 PUFA protocols and national standards for foods for special medical purposes (FSMP) for cancer patients in China, this work offers crucial data to bridge this gap and has immediate translational value for guiding clinical practice and future policy development.

Therefore, while this analysis proposes a beneficial dosage range, its precision is constrained by the available data. Future work should include prospective, multicenter RCTs specifically designed to establish a dose–response relationship, alongside mechanistic animal studies, to precisely define the optimal dosing strategy for clinical practice.

## 5. Conclusions

ω-3 PUFAs, recognized as essential dietary immunonutrients, have been widely studied for their potential to improve postoperative safety and recovery among CRC patients. This meta-analysis suggests that a daily intake of 0.16–0.31 g/kg of body weight can confer multiple clinical benefits, including enhanced immune function, attenuated systemic inflammation, fewer complications, improved quality of life, and reduced hospitalization. While these findings provide a valuable foundation for developing foods for special medical purposes (FSMPs) and guiding future research, the inherent limitations of the included studies underscore the need for further investigation. Future preclinical and clinical trials are crucial to elucidate dose–response relationships, refine optimal dosage, and longitudinally assess long-term safety. Such evidence will be pivotal for formulating evidence-based CRC management strategies that incorporate ω-3 PUFAs and for informing comprehensive clinical risk-benefit assessments.

## Figures and Tables

**Table 1:** Characteristics of the Included Randomized Controlled Trials.

First Author, Year	Research Method	Num	Age/years	Treatments	Dosages	Administration	Targets	Reference
Gao, 2011	RCT	63	E 61.5 ± 18.1 C 60.4 ± 19.5	1 W	0.1 g/(kg·d)	Venoclysis (PN)	②③⑫	[18]
Song, 2017	RCT	68	E 47–68 C 49–69	Pre 1 W	100 mL ω-3 PUFAs	venoclysis (PN)	①③④⑤⑦⑧⑨⑩	[19]
Wang, 2020	RCT	54	58–82	1 W	0.2 g/(kg·d)	venoclysis (PN)	①②③⑨⑮⑳	[20]
Qu, 2020	RCT	99	E 76.54 ± 4.36 C 75.82 ± 3.81	Pre 1 W Post 1 W	20 mL/d	PN	①②③④⑤⑥⑦⑧⑨⑩⑭⑮⑯⑰	[21]
Liang, 2008	Double-blind, RCT	41	E Avg 55.80 C Avg 59.16	Post 7 d	0.2 g/(kg·d)	fish oil	④⑤⑥⑦⑧⑨⑪	[22]
Cheng, 2016	RCT	50	46–75	Post 1 W	200 mL/d	venoclysis (PN)	②③⑫	[23]
Hu, 2018	RCT	40	E Avg 62.16 C Avg 59.13	Pre 5 d Post 1 W	10% fish oil 100 mL/d	PN	①②③④⑤⑥⑦⑨⑩㉑㉒㉓	[24]
Jiang, 2020	RCT	100	E Avg 61.83 C Avg 62.79	Pre 1 W Post 1 W	2 mL/(kg·d) fish oil	venoclysis (PN)	①②③⑨⑩⑪⑭⑯⑰	[25]
Liu, 2009	RCT	40	E Avg 54 C Avg 57	Post 1 W	10 g/d	PN	①②③④⑤⑥⑪⑭⑯⑰㉑㉒㉓	[26]
Sun, 2017	RCT	96	E Avg 60.1 C Avg 60.7	Post 1 W	2 mL/(kg·d) fish oil	PN	②③⑨⑩⑭	[27]
Teng, 2016	RCT	80	42–80	Post 1 W	10% fish oil 100 mL/d	PN	①②③④⑤⑥⑦⑬⑭⑯⑰	[28]
Zheng, 2011	RCT	40	E Avg 54.63 C Avg 56.19	Pre 1 W Post 1 W	10% fish oil 100 mL/d	PN	①②③④⑤⑦⑬㉑㉓	[29]
Zhuang, 2013	RCT	40	Avg 56.2	Post 1 W	Hourly Intervention EN	EN	③④⑤⑥⑦㉑㉒㉓	[30]
Cai, 2014	RCT	40	Avg 56.2	Post 1 W	Hourly Intervention EN	EN	③㉑㉒	[31]
Chen, 2016	RCT	97	18–70	Pre 1 W	2 mL/kg·d	PN	⑧⑨	[32]
Jiang, 2020(2)	RCT	77	E 62.81 ± 5.39 C 63.05 ± 5.27	Pre 5 d Post 1 W	10% fish oil 100 mL/d	PN	①③㉑㉒㉓	[33]
Li, 2018	RCT	150	E 58.91 ± 9.28 C 59.86 ± 9.12	Post 5 d	1–2 mL/kg·d ω-3 fish oil	PN	④⑤⑥⑭⑮⑯⑰㉑㉒㉓	[34]
Li, 2025	RCT	94	E 55.41 ± 6.82 C 55.63 ± 6.71	Post 24 W	10 mL/d ω-3 PUFA	PN	①③④⑤⑥⑧⑨⑩⑬㉑㉒㉓	[35]
Wang, 2025	RCT	126	E 63.00 ± 10.32 C 65.43 ± 10.50	Post 3 d	Enteral Nutritional Emulsion	EN	②⑧⑨⑭⑮	[36]
Wang, 2019	RCT	100	E 60.12 ± 10.14 C 60.24 ± 10.09	Post 5 d	fish oil	PN	③⑤⑥⑦⑭⑯⑰㉑㉒㉓	[37]
Zhou, 2013	RCT	38	E 54.2 ± 10.86 C 53.8 ± 11.25	Post 5 d	0.2 g/kg fish oil	PN	①②③	[38]
Sorensen, 2014	Double-blind, RCT	148	41–89	Pre 1 W Post 1 W	2.0 g/d EPA 1.0 g/d DHA	oral	⑭⑱	[39]
Hossain, 2019	Double-blind, RCT	56	61–80	Pre 5 d Post 3 W	3.0 g/d EPA	oral capsules	⑭	[40]
Cockbain, 2014	Double-blind, RCT	88	≥18	6 M	2.0 g/d EPA	oral	⑱	[41]
Courtney, 2007	Double-blind, RCT	30	E 53.1 ± 14.1 C 55.4 ± 11.5	3 M	2.0 g/d EPA	enteric-coated oral administration	⑱⑲	[42]
Camargo, 2015	Double-blind, RCT	30	E 52.1 ± 7.6 C 53.1 ± 10.2	9 W	0.36 g/d EPA 0.24 g/d DHA	fish oil	⑳	[43]
Anti, 1992	Double-blind, RCT	24	E 42–67 C 44–68	12 W	4.1 g/d EPA 3.6 g/d DHA	oral fish oil capsules	⑱	[44]
Pot, 2009	RCT	242	E1 55.1 E2 57.4 C 55.3	6 M	T1: 1.4 g/d ω-3 PUFAs T2: 0.09 g/d ω-3 PUFAs	increased fish intake	⑱	[45]
West, 2009	Double-blind, RCT	55	E Avg 39.5 C Avg 42.5	6 M	2 g/d EPA-FFA	oral capsules	⑱	[46]
Jiang, 2010	Double-blind, RCT	206	E Avg 56.3 C Avg 58.2	Post 7 d	0.2 g/kg·d fish oil	fish oil	⑤⑥⑦⑧⑨	[47]
Zhu, 2012	Double-blind, RCT	57	E 69.8 ± 10.5 C 70.8 ± 6.4	Post 8 d	0.2 g/kg·d fish oil	fish oil	⑤⑥⑦⑧⑨⑭	[48]
Murff, 2021	Double-blind, RCT	141	40–80	6 M	2.5 g/d ω-3 LCPUFAs	oral capsules	⑲	[49]
Ge, 2024	RCT	268	E Avg 36.8 C Avg 38.7	Post 1 d	10% *n*-3 PUFAs	PN	②⑧⑨⑭⑮⑯⑰	[50]
Mocellin, 2013	RCT	11	E Avg 55.2 C Avg 53.6	Post 9 W	2 g/d fish oil	oral capsules	②③⑧	[51]

Note: (1) Num: Number of patients; E: Experimental group/Observation group; C: Control group/Non-intervention group; M: month(s); W: week(s); d: day(s); EN: Enteral Nutrition; PN: Parenteral Nutrition. (2) Outcome indicators: ① TP: serum total protein; ② CRP: C-reactive protein; ③ Alb: serum albumin; ④ CD3^+^T cell (%) levels; ⑤ CD4^+^T cell (%) levels; ⑥ CD8^+^T cell (%) levels; ⑦ CD4^+^/CD8^+^T cell ratio; ⑧ TNF-α: tumor necrosis factor-α; ⑨ IL-6: interleukin-6; ⑩ PCT: procalcitonin; ⑪ WBC: white blood cell; ⑫ KPS: Karnofsky Performance Status; ⑬ TF: transcription factor; ⑭ Complications; ⑮ Length of hospitalization; ⑯ Anastomotic leak; ⑰ Incision infection; ⑱ CONC of ω-3 PUFAs; ⑲ Crypt cell proliferation; ⑳ Mortality rate; ㉑ IgA: immunoglobulin A levels; ㉒ IgG: immunoglobulin G levels; ㉓ IgM: immunoglobulin M levels.

**Table 2:** Relative Changes in Outcome Indicators Across the Included Randomized Controlled Trials.

Intervention Measures			Outcome Indicators (Change Rate)													
First Author, Year	Treatments	Dosages (g/kg·d)	TP	CRP	Alb	CD3^+^ (%)	CD4^+^ (%)	CD8^+^ (%)	CD4^+^/CD8^+^	TNF-α	IL-6	PCT	Complications	IgA	IgG	IgM
Gao, 2011	1 W	0.1	-	−25.65%	10.36%	-	-	-	-	-	-	-	-	-	-	-
Song, 2017	Pre 1 W	0.16	23.83%	-	21.31%	22.05%	22.28%	-	48.62%	1.71%	−32.26%	−57.14%	-	-	-	-
Wang, 2020	1 W	0.2	18.78%	−62.56%	42.28%	-	-	-	-	-	−45.31%	-	-	-	-	-
Qu, 2020	Pre 1 W Post 1 W	0.31	23.09%	−73.20%	34.72%	2.30%	7.55%	−4.26%	30.16%	−55.06%	−74.37%	−95.65%	−79.46%	-	-	-
Liang, 2008	Post 7 d	0.2	-	-	-	5.27%	16.22%	−1.66%	16.88%	−9.12%	−74.47%	-	-	-	-	-
Cheng, 2016	Post 1 W	0.06	-	−24.26%	9.92%	-	-	-	-	-	-	-	-	-	-	-
Hu, 2018	Pre 5 d Post 1 W	0.16	15.13%	−57.52%	24.53%	8.28%	24.91%	16.11%	7.69%	-	−43.38%	−71.74%	-	16.93%	31.19%	50.50%
Jiang, 2020	Pre 1 W Post 1 W	0.2	20.63%	−77.43%	14.50%	-	-	-	-	-	−35.57%	−75.47%	−77.78%	-	-	-
Liu, 2009	Post 1 W	0.16	−6.43%	−59.98%	7.93%	2.88%	23.79%	−10.22%	-	-	-	-	−25.00%	23.00%	6.91%	98.40%
Sun, 2017	Post 1 W	0.2	-	−72.28%	−2.06%	-	-	-	-	-	−70.72%	−32.61%	−62.50%	-	-	-
Teng, 2016	Post 1 W	0.16	20.63%	20.63%	14.27%	20.01%	19.92%	4.64%	50.00%	-	-	-	−71.43%	-	-	-
Zheng, 2011	Pre 1 W Post 1 W	0.16	1.07%	−25.50%	17.92%	7.58%	22.62%	-	12.03%	-	-	-	-	10.78%	-	13.86%
Zhuang, 2013	Post 1 W	0.06	-	-	6.65%	2.87%	10.45%	−25.44%	48.00%	-	-	-	-	20.38%	48.60%	72.50%
Jiang, 2010	Post 7 d	0.2	-	-	-	-	25.89%	−7.27%	21.28%	−6.51%	-	-	-	-	-	-
Zhu, 2012	Post 8 d	0.2	-	-	-	-	−12.10%	−11.24%	−13.33%	23.91%	2.25%	-	−50.00%	-	-	-
Cai, 2014	Post 1 W	0.2	-	-	6.65%	-	-	-	-	-	-	-	-	20.38%	48.60%	62.14%
Jiang, 2020(2)	Pre 5 d Post 1 W	0.1	15.81%	-	26.67%	-	-	-	-	-	-	-	-	34.05%	61.70%	-
Li, 2018	Post 5 d	0.04	-	-	-	6.71%	15.43%	−1.80%	-	-	-	-	−60.00%	36.80%	19.16%	16.67%
Li, 2025	Post 24 W	0.2	20.71%	-	7.30%	23.40%	12.86%	−14.66%	-	-	-	-	-	-	22.48%	10.00%
Wang, 2019	Post 5 d	0.62	-	-	9.21%	-	6.86%	−13.78%	21.19%	-	-	-	−66.67%	15.98%	19.76%	9.94%
Zhou, 2013	Post 5 d	0.2	16.82%	−77.08%	27.19%	-	-	-	-	-	-	-	-	-	-	-

Note: (1) W: week(s); d: day(s); TP: serum total protein; CRP: C-reactive protein; Alb: serum albumin; CD3^+^ (%): CD3^+^T cell (%) levels; CD4^+^ (%): CD4^+^T cell (%) levels; CD8^+^ (%): CD8^+^T cell (%) levels; CD4^+^/CD8^+^:CD4^+^/CD8^+^T cell ratio; TNF-α: tumor necrosis factor-α; IL-6: interleukin-6; PCT: procalcitonin; IgA: immunoglobulin A levels; IgG: immunoglobulin G levels; IgM: immunoglobulin M levels.

## Data Availability

The original contributions presented in the study are included in the article/Supplementary Materials. Further inquiries can be directed to corresponding authors.

## Supplementary Materials

The following supporting information can be downloaded at https://www.mdpi.com/article/10.3390/nu18010173/s1. Supplementary Date S1: PRISMA 2020 Checklist; Supplementary Date S2: Search strategy.
