# Supplementary material for: The Role of Omega-3 Polyunsaturated Fatty Acid Supplementation in Postoperative Recovery of Colorectal Cancer: Systematic Review and Meta-Analysis
Source: Nutrients. 2026 Jan 5;18(1):173. doi: 10.3390/nu18010173 (PMC12787802; doi:10.3390/nu18010173)
Supplement: Supplementary file 1 [file nutrients-18-00173-s001.zip › Supplementary Date S2.pdf]

Databa se	Search strategy
Pubmed	Search: (((((((((((((((((((((((Colonic Neoplasm[Title/Abstract]) OR (Neoplasm, Colonic[Title/Abstract])) OR (Colon Neoplasms[Title/Abstract])) OR (Colon Neoplasm[Title/Abstract])) OR (Neoplasm, Colon[Title/Abstract])) OR (Neoplasms, Colon[Title/Abstract])) OR (Neoplasms, Colonic[Title/Abstract])) OR (Cancer of Colon[Title/Abstract])) OR (Colon Cancers[Title/Abstract])) OR (Cancer of the Colon[Title/Abstract])) OR (Colonic Cancer[Title/Abstract])) OR (Cancer, Colonic[Title/Abstract])) OR (Cancers, Colonic[Title/Abstract])) OR (Colonic Cancers[Title/Abstract])) OR (Colon Cancer[Title/Abstract])) OR (Cancer, Colon[Title/Abstract])) OR (Cancers, Colon[Title/Abstract])) OR (Colon Adenocarcinoma[Title/Abstract])) OR (Adenocarcinoma, Colon[Title/Abstract])) OR (Adenocarcinomas, Colon[Title/Abstract])) OR (Colon Adenocarcinomas[Title/Abstract])) OR ("Colonic Neoplasms"[Mesh])) AND (((((((((((((((((((((((((((((((N-3 Fatty Acid[Title/Abstract]) OR (Acid, N-3 Fatty[Title/Abstract])) OR (Fatty Acid, N-3[Title/Abstract])) OR (N 3 Fatty Acid[Title/Abstract])) OR (Omega-3 Fatty Acid[Title/Abstract])) OR (Acid, Omega-3 Fatty[Title/Abstract])) OR (Fatty Acid, Omega-3[Title/Abstract])) OR (Omega 3 Fatty Acid[Title/Abstract])) OR (Omega-3 Fatty Acids[Title/Abstract])) OR (n-3 Oil[Title/Abstract])) OR (n 3 Oil[Title/Abstract])) OR (Oil, n-3[Title/Abstract])) OR (n3 Oil[Title/Abstract])) OR (Oil, n3[Title/Abstract])) OR (n-3 Fatty Acids[Title/Abstract])) OR (n 3 Fatty Acids[Title/Abstract])) OR (n-3 Polyunsaturated Fatty Acid[Title/Abstract])) OR (n 3 Polyunsaturated Fatty Acid[Title/Abstract])) OR (n-3 PUFA[Title/Abstract])) OR (n 3 PUFA[Title/Abstract])) OR (PUFA, n-3[Title/Abstract])) OR (n3 Fatty Acid[Title/Abstract])) OR (Fatty Acid, n3[Title/Abstract])) OR (n3 PUFA[Title/Abstract])) OR (PUFA, n3[Title/Abstract])) OR (n3 Polyunsaturated Fatty Acid[Title/Abstract])) OR (n3 Oils[Title/Abstract])) OR (Omega 3 Fatty Acids[Title/Abstract])) OR (n-3 Oils[Title/Abstract])) OR (n 3 Oils[Title/Abstract])) OR ("Fatty Acids, Omega-3"[Mesh]))) AND (randomized controlled trial[Publication Type] OR randomized[Title/Abstract] OR placebo[Title/Abstract])
Embase	#1 'colorectal cancer'/exp OR 'colorectal cancer'<br>#2 'cancer of colon and rectum' OR 'cancer of rectum and col

	on' OR 'cancer of the colon and rectum' OR 'cancer of the colon and the rectum' OR 'cancer of the rectum and colon' OR 'cancer of the rectum and the colon' OR 'colo-rectal cancer' OR 'colo-rectal carcinogenesis' OR 'colo-rectal malignancies' OR 'colo-rectal malignancy' OR 'colorectal cancerogenesis' OR 'colorectal carcinogenesis' OR 'colorectal malignancies' OR 'colorectal malignancy' OR 'malignancies of the colon and rectum' OR 'malignancy of colon and rectum' OR 'malignancy of the colon and rectum' OR 'recto-colonic cancer' OR 'rectocolonic cancer' OR 'colorectal cancer'<br>#3 #1 OR #2<br>#4 'omega 3 fatty acid'/exp<br>#5 'bilantin omega' OR 'conchol 36' OR 'eicosa e' OR 'eicosapen' OR 'epaisdin' OR 'epanova' OR 'fatty acids, omega 3' OR 'fatty acids, omega-3' OR 'n 3 fatty acid' OR 'n 3 polyunsaturated fatty acid' OR 'omega 3' OR 'omega 3 carboxylic acid' OR 'omega 3 carboxylic acids' OR 'omega 3 feingold' OR 'omega 3 plus' OR 'omega 3 polyunsaturated fatty acid' OR 'omega forte' OR 'omega-3-carboxylic acids' OR 'omega3 polyunsaturated fatty acid' OR 'sakana' OR 'sanhelios omega 3' OR 'omega 3 fatty acid'<br>#6 #4 OR #5<br>#7 'fish oil'/exp OR 'fish oil'<br>#8 'ameu' OR 'efamed' OR 'epax' OR 'epax 5000' OR 'feniko' OR 'fish oils' OR 'himega' OR 'k 85' OR 'k 85 fish oil preparation' OR 'lachs 550' OR 'lipitac' OR 'maxepa' OR 'olemar' OR 'omegaven' OR 'optimepa' OR 'pikasol' OR 'promega' OR 'super epa' OR 'superepa' OR 'tuna oil' OR 'fish oil'<br>#9 #7 OR #8<br>#10 'docosahexaenoic acid'/exp OR 'docosahexaenoic acid'<br>#11 'dhasco' OR 'docosahexaenoate' OR 'docosahexaenoic acids' OR 'docosahexenoic acid' OR 'docosahexaenoic acid'<br>#12 #10 OR #11<br>#13 'icosapentaenoic acid'/exp OR 'icosapentaenoic acid'<br>#14 '5, 8, 11, 14, 17 eicosapentaenoic acid' OR '5, 8, 11, 14, 17 icosapentaenoic acid' OR '5, 8, 11, 14, 17-eicosapentaenoic acid' OR 'eicosa 5, 8, 11, 14, 17 pentaene carboxylic acid' OR 'eicosa 5, 8, 11, 14, 17 pentaenoic acid' OR 'eicosapentaenoate' OR '

	eicosapentaenoic acid' OR 'eicosapentenoic acid' OR 'epaspire' OR 'icosa 5, 8, 11, 14, 17 pentaenoic acid' OR 'icosapent' OR 'icosapentaenoate' OR 'omega 3 eicosapentaenoic acid' OR 'timnodonate' OR 'timnodonic acid' OR 'icosapentaenoic acid'<br>#15 #13 OR #14<br>#16 'randomized controlled trial (topic)'/exp OR 'randomized controlled trial (topic)'<br>#17 'pragmatic clinical trials as topic' OR 'randomized controlled trials' OR 'randomized controlled trials as topic' OR 'randomized controlled trial (topic)'<br>#18 #16 OR #17<br>#19 #6 OR #9 OR #12 OR #15<br>#20 #3 AND #18 AND #19
Web of Science	1 "TS=(Colonic Neoplasms) and Preprint Citation Index (Exclude – Database) "<br>2 "((((((((((((((((((TS=(Colonic Neoplasm)) OR TS=(Neoplasm, Colonic)) OR TS=(Colon<br>Neoplasms)) OR TS=(Colon Neoplasm)) OR TS=(Neoplasm, Colon)) OR TS=(Neoplasms,<br>Colon)) OR TS=(Neoplasms, Colonic)) OR TS=(Cancer of Colon)) OR TS=(Colon Cancers)) OR<br>TS=(Cancer of the Colon)) OR TS=(Colonic Cancer)) OR TS=(Cancer, Colonic)) OR<br>TS=(Cancers, Colonic)) OR TS=(Colonic Cancers)) OR TS=(Colon Cancer)) OR TS=(Cancer,<br>Colon)) OR TS=(Cancers, Colon)) OR TS=(Colon Adenocarcinoma)) OR TS=(Adenocarcinoma,<br>Colon)) OR TS=(Adenocarcinomas, Colon)) OR TS=(Colon Adenocarcinomas) and Preprint Citation Index (Exclude – Database) "<br>3 "#2 OR #1 and Preprint Citation Index (Exclude – Database) "<br>4 "TS=(N-3 Fatty Acid) and Preprint Citation Index (Exclude – Database) "<br>5 "((((((((((((((((((((((((((TS=(Acid, N-3 Fatty)) OR TS=(Fatty Acid, N-3)) OR TS=(N 3 Fatty Acid))

	OR TS=(Omega-3 Fatty Acid)) OR TS=(Acid, Omega-3 Fatty)) OR TS=(Fatty Acid, Omega-3)) OR TS=(Omega 3 Fatty Acid)) OR TS=(Omega-3 Fatty Acids)) OR TS=(n-3 Oil)) OR TS=(n 3Oil)) OR TS=(Oil, n-3)) OR TS=(n3 Oil)) OR TS=(Oil, n3)) OR TS=(n-3 Fatty Acids)) OR TS=(n 3 Fatty Acids)) OR TS=(n-3 Polyunsaturated Fatty Acid)) OR TS=(n 3 Polyunsaturated Fatty Acid)) OR TS=(n-3 PUFA)) OR TS=(n 3 PUFA)) OR TS=(PUFA, n-3)) OR TS=(n3 Fatty Acid)) OR TS=(Fatty Acid, n3)) OR TS=(n3 PUFA)) OR TS=(PUFA, n3)) OR TS=(n3 Polyunsaturated Fatty Acid)) OR TS=(n3 Oils)) OR TS=(Omega 3 Fatty Acids)) OR TS=(n-3 Oils)) OR TS=(n 3 Oils) and Preprint Citation Index (Exclude – Database) "<br>6 "#4 OR #5 and Preprint Citation Index (Exclude – Database) "<br>7 "TS=(Fish Oils) and Preprint Citation Index (Exclude – Database) "<br>8 "(((((TS=(Oils, Fish)) OR TS=(Fish Oil)) OR TS=(Oil, Fish)) OR TS=(Fish Liver Oils)) OR TS=(Liver Oils, Fish)) OR TS=(Oils, Fish Liver) and Preprint Citation Index (Exclude – Database) "<br>9 "#7 OR #8 and Preprint Citation Index (Exclude – Database) "<br>10 "TS=(Docosahexaenoic Acids) and Preprint Citation Index (Exclude – Database) "<br>11 "((((((((((((((((TS=(Acids, Docosahexaenoic)) OR TS=(Docosahexenoic Acids)) OR TS=(Acids, Docosahexenoic)) OR TS=(Docosahexaenoic Acid)) OR TS=(Acid, Docosahexaenoic)) OR TS=(Docosahexaenoic Acid (All-Z Isomer))) OR TS=(Docosahexaenoic Acid, 4,7,10,13,16,19-(All-Z-Isomer))) OR TS=(Docosahexaenoic Acid, 4,7,10,13,16,19-Isomer, Sodium Salt)) OR TS=(Docosahexaenoic Acid, 3,6,9,12,15,18-Isomer)) OR TS=(Docosahexaenoic Acid, Sodium Salt)) OR TS=(Docosahexaenoic Acid, 4,7,10,13,16,19-Isomer))) OR TS=(Docosahexaenoic Acid, 4,7,10,13,16,19-(All-Z-Isomer), Potassium Salt)) OR TS=(Docosahexaenoic Acid Dimer (All-Z Isomer))) OR

	TS=(Docosahexaenoic Acid, 4,7,10,13,16,19-(All-Z-Isomer), Cesium Salt)) OR TS=(Docosahexaenoic Acid, 4,7,10,13,16,19-(All-Z-Isomer), Cerium Salt)) OR TS=(Docosahexaenoate)) OR TS=(Docosahexaenoic Acid, 4,7,10,13,16,19-(Z,Z,Z,Z,Z,E-Isomer)) and Preprint Citation Index (Exclude – Database) "<br>12 "#11 OR #10 and Preprint Citation Index (Exclude – Database) "<br>13 "TS=(Eicosapentaenoic Acid) and Preprint Citation Index (Exclude – Database) "<br>14 "(((((((TS=(Icosapent)) OR TS=(5,8,11,14,17-Eicosapentaenoic Acid)) OR TS=(5,8,11,14,17-Icosapentaenoic Acid)) OR TS=(omega-3-Eicosapentaenoic Acid)) OR TS=(omega 3 Eicosapentaenoic Acid)) OR TS=(Timnodonic Acid)) OR TS=(Eicosapentanoic Acid)) OR TS=(Acid, Eicosapentanoic) and Preprint Citation Index (Exclude – Database) "<br>15 "#13 OR #14 and Preprint Citation Index (Exclude – Database) "<br>16 "#15 OR #12 OR #9 OR #6 and Preprint Citation Index (Exclude – Database) "<br>17 "(((AB=(randomized controlled trial)) OR AB=(randomized)) OR AB=(placebo)) OR AB=(RCT) and Preprint Citation Index (Exclude – Database) "<br>18 "#16 AND #17 AND #3 and Preprint Citation Index (Exclude – Database) "
The Cochrane Library	#1 MeSH descriptor: [Colonic Neoplasms] explode all trees<br>#2 (Colonic Neoplasm):ti,ab,kw OR (Neoplasm, Colonic):ti,ab,kw OR (Colon Neoplasms):ti,ab,kw OR (Colon Neoplasm):ti,ab,kw OR (Neoplasm, Colon):ti,ab,kw OR (Neoplasms, Colon):ti,ab,kw OR (Neoplasms, Colonic):ti,ab,kw OR (Cancer of Colon):ti,ab,kw OR (Colon Cancers):ti,ab,kw OR (Cancer of the Colon):ti,ab,kw OR (Colonic Cancer):ti,ab,kw OR (Cancer, Colonic):ti,ab,kw OR (Cancers, Colonic):ti,ab,kw OR (Colonic Cancers):ti,ab,kw OR (Colon Cancer):ti,ab,kw OR (Cancer, Colon):ti,ab,kw OR (Cancers, Colon):ti,ab,kw OR (Colon Adenocarcinoma):ti,ab,kw OR (Adenocarcinoma, Colon):ti,ab,kw OR (Adenocarcinomas, Colon):ti,ab,kw OR (Colon Adenocarcinomas):ti,ab,kw

	#3 #1 OR #2<br>#4 MeSH descriptor: [Fatty Acids, Omega-3] explode all trees<br>#5 (Acid, N-3 Fatty):ti,ab,kw OR (Fatty Acid, N-3):ti,ab,kw OR (N 3 Fatty Acid):ti,ab,kw OR (Omega-3 Fatty Acid):ti,ab,kw OR (Acid, Omega-3 Fatty):ti,ab,kw OR (Fatty Acid, Omega-3):ti,ab,kw OR (Omega 3 Fatty Acid):ti,ab,kw OR (Omega-3 Fatty Acids):ti,ab,kw OR (n-3 Oil):ti,ab,kw OR (n 3 Oil):ti,ab,kw OR (Oil, n-3):ti,ab,kw OR (n3 Oil):ti,ab,kw OR (Oil, n3):ti,ab,kw OR (n-3 Fatty Acids):ti,ab,kw OR (n 3 Fatty Acids):ti,ab,kw OR (n-3 Polyunsaturated Fatty Acid):ti,ab,kw OR (n 3 Polyunsaturated Fatty Acid):ti,ab,kw OR (n-3 PUFA):ti,ab,kw OR (n 3 PUFA):ti,ab,kw OR (PUFA, n-3):ti,ab,kw OR (n3 Fatty Acid):ti,ab,kw OR (Fatty Acid, n3):ti,ab,kw OR (n3 PUFA):ti,ab,kw OR (PUFA, n3):ti,ab,kw OR (n3 Polyunsaturated Fatty Acid):ti,ab,kw OR (n3 Oils):ti,ab,kw OR (Omega 3 Fatty Acids):ti,ab,kw OR (n-3 Oils):ti,ab,kw OR (n 3 Oils):ti,ab,kw<br>#6 #4 OR #5<br>#7 MeSH descriptor: [Fish Oils] explode all trees 4649<br>#8 (Oils, Fish):ti,ab,kw OR (Fish Oil):ti,ab,kw OR (Oil, Fish):ti,ab,kw OR (Fish Liver Oils):ti,ab,kw OR (Liver Oils, Fish):ti,ab,kw OR (Oils, Fish Liver):ti,ab,kw<br>#9 #7 OR #8<br>#10 MeSH descriptor: [Docosahexaenoic Acids] explode all trees<br>#11 ("Docosahexaenoic Acid, 4,7,10,13,16,19-(All-Z-Isomer), Potassium Salt" OR "Docosahexaenoic Acid, 4,7,10,13,16,19-(All-Z-Isomer), Cerium Salt" OR "Docosahexaenoic Acid, Sodium Salt" OR "Docosahexaenoic Acid, 4,7,10,13,16,19-(All-Z-Isomer), Cesium Salt" OR "Docosahexaenoic Acid Dimer (All-Z Isomer)" OR "Docosahexaenoic Acid, 4,7,10,13,16,19-Isomer" OR "Docosahexaenoic Acid, 3,6,9,12,15,18-Isomer" OR Docosahexaenoate OR "Docosahexaenoic Acid (All-Z Isomer)" OR "Docosahexaenoic Acid, 4,7,10,13,16,19-(All-Z-Isomer)" OR "Docosahexaenoic Acid, 4,7,10,13,16,19-(Z,Z,Z,Z,Z,E-Isomer)" OR "Acids, Docosahexaenoic" OR "Acid, Docosahexaenoic" OR "Docosahexaenoic Acid" OR "Acids, Docosahexenoic" OR "Docosahexenoic Acids" OR "Docosahexaenoic Acid, 4,7,10,13,16,19-Isomer, Sodium Salt"):ti,ab,kw

	#12 #10 OR #11<br>#13 MeSH descriptor: [Eicosapentaenoic Acid] explode all trees<br><br>#14 (Icosapent OR "5,8,11,14,17-Eicosapentaenoic Acid" OR "5,8,11,14,17-Icosapentaenoic Acid" OR "omega-3-Eicosapentaenoic Acid" OR "omega 3 Eicosapentaenoic Acid" OR "Timnodonic Acid" OR "Eicosapentanoic Acid" OR "Acid, Eicosapentanoic"):ti,ab,kw<br>#15 #13 OR #14<br>#16 #6 OR #9 OR #12 OR #15<br>#17 (randomized controlled trial):ti,ab,kw OR (randomized):ti,ab,kw OR (placebo):ti,ab,kw OR (RCT):ti,ab,kw<br>#18 #3 AND #16<br>#19 #18 AND #17
CNKI	(SU='结肠癌' OR SU='结直肠癌' OR SU='结直肠肿瘤' OR SU='结肠肿瘤' OR SU='结肠恶性肿瘤' OR SU='结直肠恶性肿瘤')<br>AND<br>(SU='Omega-3不饱和脂肪酸' OR SU='Omega-3多不饱和脂肪酸' OR SU='Omega-3脂肪酸' OR SU='n-3脂肪酸' OR SU='n-3不饱和脂肪酸' OR SU='n-3多不饱和脂肪酸' OR SU='ω–3脂肪酸' OR SU='ω–3不饱和脂肪酸' OR SU='ω–3多不饱和脂肪酸' OR SU='鱼油' OR SU='鱼油脂肪乳' OR SU='鱼油脂肪乳剂' OR SU='鱼油制剂' OR SU='精制鱼油' OR SU='ω-3鱼油' OR SU='ω-3鱼油脂肪乳' OR SU='藻油' OR SU='藻油提取' OR SU='亚麻籽油' OR SU='二十碳五烯酸' OR SU='EPA' OR SU='Eicosapentaenoic Acid' OR SU='DHA' OR SU='二十二碳六烯酸' OR SU='Docosahexaenoic Acid' OR SU='α-亚麻酸' OR SU='ALA')<br>AND<br>((TKA='随机对照试验'% OR TKA='随机对照'% OR TKA='随机'% OR TKA='RCT'%))
VIP	(M=("结肠癌" OR "结直肠癌" OR "结直肠肿瘤" OR "结肠肿瘤" OR "结肠恶性肿瘤" OR "结直肠恶性肿瘤"))<br>AND<br>(M=("Omega-3不饱和脂肪酸" OR "Omega-3多不饱和脂肪酸" OR "Omega-3脂肪酸" OR "n-3脂肪酸" OR "n-3不饱和脂肪酸" OR "n-3多不饱和脂肪酸" OR "ω–3脂肪酸" OR "ω–3不饱和脂肪酸" OR "ω–3多不饱和脂肪酸" OR "鱼油" OR "鱼油脂肪乳" OR "鱼油脂肪乳剂" OR "

	鱼油制剂" OR "精制鱼油" OR "ω-3鱼油" OR "ω-3鱼油脂肪乳" OR "藻油" OR "藻油提取" OR "亚麻籽油" OR "二十碳五烯酸" OR "EPA" OR "Eicosapentaenoic Acid" OR "DHA" OR "二十二碳六烯酸" OR "Docosahexaenoic Acid" OR "α-亚麻酸" OR "ALA"))<br>AND<br>((R="随机对照试验" OR R="随机对照" OR R="随机" OR R="RCT"))
CBM	1) "结肠肿瘤"[不加权:扩展]<br>2) "结肠恶性肿瘤"[常用字段:智能]<br>3) "结直肠癌"[常用字段:智能]<br>4) "结直肠肿瘤"[常用字段:智能]<br>5) "结肠癌"[常用字段:智能]<br>6) "结直肠恶性肿瘤"[常用字段:智能]<br>7) (((((((((#1) OR (#2))) OR (#3))) OR (#4))) OR (#5))) OR (#6))<br>8) "脂肪酸类，ω3"[不加权:扩展]<br>9) "n-3脂肪酸类"[常用字段:智能]<br>10) "ω-3脂肪酸类"[常用字段:智能]<br>11) "n-3脂肪酸"[常用字段:智能]<br>12) "n-3多不饱和脂肪酸"[常用字段:智能]<br>13) "n-3PUFA"[常用字段:智能]<br>14) "二十碳五烯酸"[不加权:扩展]<br>15) "EPA"[常用字段:智能]<br>16) "二十二碳六烯酸类"[不加权:扩展]<br>17) "DHA"[常用字段:智能]<br>18) "鱼油"[不加权:扩展]<br>19) "精制鱼油"[常用字段:智能]<br>20) "鱼油脂肪乳"[常用字段:智能]<br>21) "鱼油制剂"[常用字段:智能]<br>22) (((((((((((((((((((((((((((#8) OR (#9))) OR (#10))) OR (#11))) OR (#12))) OR (#13))) OR (#14))) OR (#15))) OR (#16))) OR (#17))) OR (#18))) OR (#19))) OR (#20))) OR (#21))<br>23) "随机对照试验"[不加权:扩展]<br>24) "随机对照"[常用字段:智能]<br>25) "随机"[常用字段:智能]<br>26) "RCT"[常用字段:智能]<br>27) (#26) OR (#25) OR (#24) OR (#23)

	28) (#27) AND (#22) AND (#7)
WanFangDate	(主题:(结肠癌 OR 结直肠癌 OR 结直肠肿瘤 OR 结肠肿瘤 OR 结肠恶性肿瘤 OR 结直肠恶性肿瘤))<br>AND<br>(主题:(Omega-3不饱和脂肪酸 OR Omega-3多不饱和脂肪酸 OR Omega-3脂肪酸 OR n-3脂肪酸 OR n-3不饱和脂肪酸 OR n-3多不饱和脂肪酸 OR ω−3脂肪酸 OR ω−3不饱和脂肪酸 OR ω−3多不饱和脂肪酸 OR 鱼油 OR 鱼油脂肪乳 OR 鱼油脂肪乳剂 OR 鱼油制剂 OR 精制鱼油 OR ω-3鱼油 OR ω-3鱼油脂肪乳 OR 藻油 OR 藻油提取 OR 亚麻籽油 OR 二十碳五烯酸 OR EPA OR Eicosapentaenoic Acid OR DHA OR 二十二碳六烯酸 OR Docosahexaenoic Acid OR α-亚麻酸 OR ALA))<br>AND<br>(全部:("随机对照试验" OR "随机对照" OR "随机" OR "RCT"))

Table S1. Search strategy
